# Sub-thermionic, ultra-high-gain organic transistors and circuits

**DOI:** 10.1038/s41467-021-22192-2

**Published:** 2021-03-26

**Authors:** Zhongzhong Luo, Boyu Peng, Junpeng Zeng, Zhihao Yu, Ying Zhao, Jun Xie, Rongfang Lan, Zhong Ma, Lijia Pan, Ke Cao, Yang Lu, Daowei He, Hongkai Ning, Wanqing Meng, Yang Yang, Xiaoqing Chen, Weisheng Li, Jiawei Wang, Danfeng Pan, Xuecou Tu, Wenxing Huo, Xian Huang, Dongquan Shi, Ling Li, Ming Liu, Yi Shi, Xue Feng, Paddy K. L. Chan, Xinran Wang

**Affiliations:** 1 National Laboratory of Solid-State Microstructures, School of Electronic Science and Engineering and Collaborative Innovation Center of Advanced Microstructures, Nanjing University, Nanjing, 210093 China; 2 Department of Mechanical Engineering, The University of Hongkong, Pok Fu Lam Road, Hong Kong, China; 3 MOE Key Laboratory of Macromolecular Synthesis and Functionalization, Department of Polymer Science and Engineering, Zhejiang University, Hangzhou, 310027 China; 4 College of Electronic and Optical Engineering, Nanjing University of Posts and Telecommunications, Nanjing, 210023 China; 5 Key Laboratory of Microelectronics Devices and Integrated Technology, Institute of Microelectronics, Chinese Academy of Sciences, Beijing, 100029 China; 6 Department of Cardiology, Nanjing Drum Tower Hospital, The Affiliated Hospital of Nanjing University Medical School, Nanjing, 210093 China; 7 Department of Mechanical Engineering, City University of Hong Kong, Kowloon, Hong Kong, China; 8 Microfabrication and Integration Technology Center, Nanjing University, Nanjing, 210093 China; 9 Department of Biomedical Engineering, Tianjin University, Tianjin, 300072 China; 10 Department of Sports Medicine and Adult Reconstructive Surgery, Nanjing Drum Tower Hospital, The Affiliated Hospital of Nanjing University Medical School, Nanjing, 210093 China; 11 AML, Department of Engineering Mechanics, Center for Flexible Electronics Technology, Tsinghua University, Beijing, 100084 China; 12 Advanced Biomedical Instrumentation Centre, Hong Kong Science Park, Shatin, New Territories, Hong Kong, China

**Keywords:** Biomedical engineering, Electronic devices, Molecular electronics

## Abstract

The development of organic thin-film transistors (OTFTs) with low power consumption and high gain will advance many flexible electronics. Here, by combining solution-processed monolayer organic crystal, ferroelectric HfZrO_x_ gating and van der Waals fabrication, we realize flexible OTFTs that simultaneously deliver high transconductance and sub-60 mV/dec switching, under one-volt operating voltage. The overall optimization of transconductance, subthreshold swing and output resistance leads to transistor intrinsic gain and amplifier voltage gain over 5.3 × 10^4^ and 1.1 × 10^4^, respectively, which outperform existing technologies using organics, oxides and low-dimensional nanomaterials. We further demonstrate battery-powered, integrated wearable electrocardiogram (ECG) and pulse sensors that can amplify human physiological signal by 900 times with high fidelity. The sensors are capable of detecting weak ECG waves (undetectable even by clinical equipment) and diagnosing arrhythmia and atrial fibrillation. Our sub-thermionic OTFT is promising for battery/wireless powered yet performance demanding applications such as electronic skins and radio-frequency identification tags, among many others.

## Abstract (web summary)

Exploiting negative capacitance effects in organic thin-film transistors (OTFTs) is advantageous for enhancing device performance. Here, the authors report solution-processed sub-thermionic OTFTs and circuits with ferroelectric hafnium oxides that show ultra-low power and ultra-high gain.

## Introduction

Organic thin-film transistors (OTFTs) have been extensively pursued for printable and flexible electronic applications owing to their intrinsic flexibility and low-cost processing^1–6^. Many applications, such as Internet of Things (IoT) and wearable electronics, require low-voltage operation while providing high enough current to drive the circuits or high gain to amplify small signals. So far, however, there exist considerable challenges to make OTFT competitive over carbon nanotube films^7^, two-dimensional (2D) materials^8^ and oxides^9^. First, the mobility of organic semiconductors is generally lower than their inorganic counterparts. This leads to low transconductance (*g*_m_) and intrinsic gain ($A_{\mathrm{i}} \,=\, g_{\mathrm{m}} \cdot r_0$, where *r*_0_ is output resistance). Second, the contact resistance, which partly arises the vertical access resistance from the finite film thickness and disorders introduced by conventional fabrication processes (such as lithography and metal deposition), is a major limiting factor towards high-frequency operation^10^. Third, the switching of OTFTs is often far from ideal, which results in large operating voltage (*V*_dd_)^11^. Despite significant efforts, it remains a challenge to maintain subthreshold swing (*SS*) close to the Boltzmann thermionic limit ($ln\left( {10} \right)\frac{{k_BT}}{q} \approx 60\,{\mathrm{mV}}/{\mathrm{dec}}$, where *q* is elementary charge, *k*_B_ is Boltzmann’s constant, and *T* is temperature) over extended range and *V*_dd_ low enough for complete battery/wireless operation^12–14^.

Recently, negative capacitance (NC) effect from ferroelectric hafnium oxides provides a promising solution for low-power and complementary metal-oxide-semiconductor (CMOS) compatible electronics^15–24^. Experimentally, NC transistors have been implemented on silicon^25–27^, germanium^28^ and 2D materials^29,30^, showing evidence of sub-60 mV/dec switching and enhanced *g*_m_. Moreover, a transition from negative to positive drain-induced barrier lowing (DIBL) was theoretically predicted and experimentally observed in NC transistors^16,29^. This would lead to infinite *r*_0_ and intrinsic gain near the transition region, which is desirable for high-gain analog amplifiers. However, this unique advantage of NC transistors has not been fully exploited so far.

Here, we combine solution-processed OTFT with ferroelectric HfZrO_x_ (HZO) gating to demonstrate flexible sub-thermionic organic transistors as well as ultra-high-gain amplifier circuits. Our technology has several unique features. First, the monolayer organic crystal film ensures excellent gate control while maintaining high channel mobility up to 10.4 cm^2^ V^−1^ s^−1^. Second, the double-well energy landscape of ferroelectric gating and associated NC effect break the thermionic limit in *SS* (60 mV/dec) and transconductance efficiency (38.7 S/A) and simultaneously enhances on-current and *g*_m_ by saving overdrive voltage loss^15,23,29,30^. In addition, the negative DIBL introduced by ferroelectric HZO^16,29^ produces large *r*_0_ ~10^10^ Ω. Third, the uniform film morphology and van der Waals (vdW) integration of metal electrodes^31^ ensure direct and damage-free contact with the channel, giving contact resistance below 60 Ωcm. Together, these systematic optimizations not only significant improve the performance and power consumption of OTFTs, but also deliver the highest gain for any TFT technologies. Finally, our sub-thermionic OTFTs are low-temperature solution processed, scalable to large area and compatible with flexible substrates, which allow us to fabricate integrated wearable sensors health monitoring and diagnosis.

## Results

### Device fabrication and characterizations

**Fig. 1:**
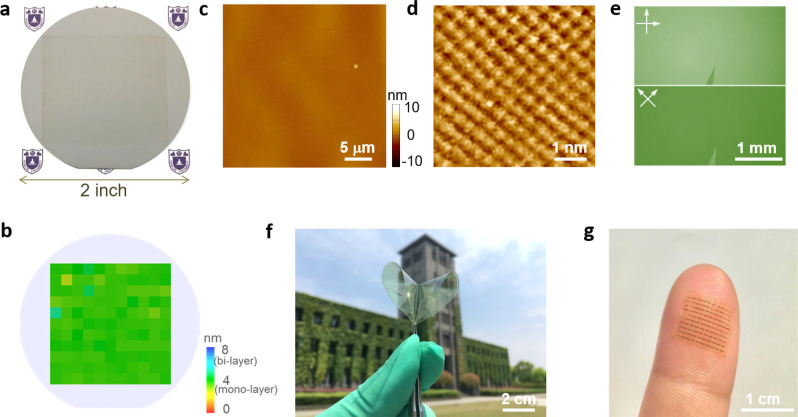
Large-area monolayer C_10_-DNTT films. **a** Photograph of uniform monolayer C_10_-DNTT film on 2-in. Si wafer. **b** The thickness mapping of the sample in **a** using spectral ellipsometry. AFM (**c**), high-resolution AFM (**d**) and cross-polarized optical micrograph (**e**) of the monolayer C_10_-DNTT film. Photograph of monolayer C_10_-DNTT film on polyimide substrate (**f**) and flexible device array laminated on a fingertip (**g**).

For device fabrication, we first grew HZO/Al_2_O_3_ gate dielectrics on highly doped silicon wafers by atomic layer deposition (ALD) and carried out solution shearing of p-type 2,9-didecyldinaphtho[2,3-b:2′,3′-f]thieno[3,2-b]thiophene (C_10_-DNTT) as channel material^32,33^ (see “Methods” section for experimental details). The Hf:Zr atomic ratio was close to 1:1 as revealed by X-ray photoelectron spectroscopy (Supplementary Fig. 1b). The HZO showed low surface roughness (~0.3 nm) and ferroelectric properties as confirmed by atomic force microscopy (AFM), piezoelectric force microscopy, and polarization-electric field (P-E) loop measurements (Supplementary Fig. 1). Highly uniform monolayer organic films were obtained by optimizing the substrate temperature and blade speed in the shearing process^34^ (Supplementary Fig. 2). Figure 1a, f show optical images of monolayer C_10_-DNTT films on 2-in. silicon wafer and polyimide, respectively. The film thickness was characterized by ellipsometry and AFM, both showing ~4 nm (corresponding to monolayer) with excellent wafer-scale uniformity (Fig. 1b, c). The films were highly crystalline with herringbone lattice, consistent with C_10_-DNTT bulk crystals (Fig. 1d). Under optimized conditions, the domain size could reach several millimeters (Fig. 1e), which effectively reduced domain boundaries within device channel. The average field-effect mobility of monolayer OTFTs on Al_2_O_3_ dielectrics was 8.44 ± 1.61 cm^2^ V^−1^ s^−1^ (Supplementary Fig. 3), further proving the high quality of C_10_-DNTT films. On ultra-thin polyimide substrate, the monolayer film was highly transparent (>97%) and conformable to epidermis to allow the construction of skin-like wearable devices (Fig. 1g, Supplementary Figs. 2b and 4).

**Fig. 2:**
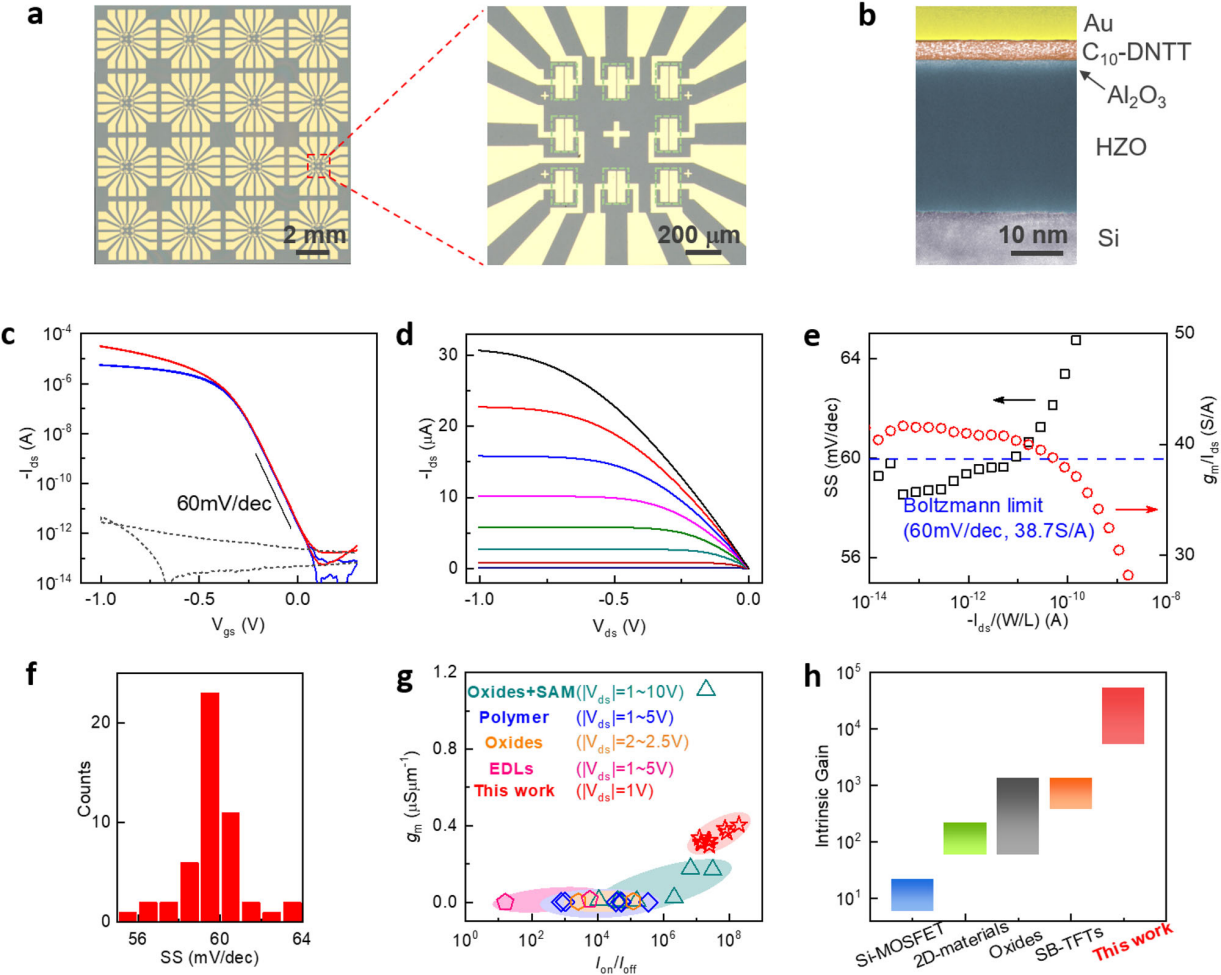
Transistor characteristics of sub-thermionic monolayer OTFTs. **a** Photographs of device arrays fabricated on silicon substrate. The length to width ratio (*L*/*W*) of device channel is 5 μm/180 μm. **b** False-color cross-section TEM image of a device contact region, clearly showing the Au/C_10_-DNTT/Al_2_O_3_/HZO/Si stack. **c** Double-sweep *I*_ds_ − *V*_gs_ curves of the sub-thermionic OTFT at *V*_ds_ = −0.1 V (blue) and −1 V (red). No hysteresis is observed. The black-dashed line is the gate leakage current. **d** *I*_ds_ − *V*_ds_ curves of the sub-thermionic OTFT. From bottom to top, *V*_gs_ is from −0.3 to −1 V. **e** *SS* (black) and transconductance efficiency (red) as a function of *I*_ds_ for the device in **c**. The Boltzmann limit reference corresponds to 300 K under which our measurements were taken. **f** Statistical distributions of one-decade *SS* for 50 devices. **g** Comparison of *I*_on_/*I*_off_ and normalized transconductance at |*V*_gs(on)_ − *V*_gs(off)_| = 1 V among OTFT technologies using different dielectrics. The extracted data are listed in Supplementary Table 2. **h** Comparison of intrinsic gain among different TFT technologies.

It is well-known that conventional OTFT fabrication processes can damage the organic films, especially for monolayer^35^. To this end, we developed solvent-free and low-energy vdW fabrication process as illustrated in Supplementary Fig. 5. This approach is readily scalable to wafer-scale with nearly 100% yield and micrometer resolution (Fig. 2a). Briefly, we first prepared patterned Au contact electrodes on a silicon wafer. The electrodes were mechanically released using PMMA and thermal release tape and gently laminated onto the pre-patterned channel areas. Such low-energy process preserved the integrity of monolayer C_10_-DNTT channel and contact interface as shown by the cross-sectional transmission electron microscopy (TEM) image (Fig. 2b). After removing the tape, electron-beam lithography (EBL) was performed to remove the PMMA on probing pads for electrical measurements. The intimate and damage-free contact to the monolayer channel indeed led to contact resistance of 59.4 ± 5.3 Ωcm as measured by transfer length method (Supplementary Fig. 6), close to the record for OTFTs (Supplementary Table 1).

### Electrical performance of sub-thermionic OTFTs and device modeling

Figure 2c, d presents the room temperature transfer (*I*_ds_ − *V*_gs_) and output (*I*_ds_ *−* *V*_ds_) characteristics of a typical sub-thermionic monolayer OTFT under *V*_dd_ = 1 V. We observed several remarkable features, including extended subthreshold region with a steep slope, close-to-zero threshold voltage (*V*_th_), on/off ratio over 10^8^, excellent saturation behavior, and no hysteresis. The derived point *SS* was below 60 mV/dec for more than two decades (Fig. 2e). The average *SS* was 58.6 and 60.9 mV/dec for one and five decades of *I*_ds_, respectively. The device also delivered superior on-state performance. In particular, the normalized *g*_m_ reached high value of 0.41 μSμm^−1^ under one-volt operation (Supplementary Table 2), suggesting possible radio-frequency applications^10^. More importantly, the transconductance efficiency, $\frac{{g_m}}{{I_{ds}}} \,=\, 41.6$ S/A, exceeded the theoretical limit of $\frac{q}{{k_BT}} \,=\, 38.7$ S/A^4^ imposed by Boltzmann distribution of carriers (Fig. 2e). The high transconductance efficiency was maintained over several orders of *I*_ds_, which was desirable in analog circuit design but difficult for CMOS^36^. To evaluate the device variations, we measured 50 OTFTs (the transfer characteristics and statistics are shown in Supplementary Fig. 7). Figure 2f presents the histogram of *SS* (over one decade of *I*_ds_ rather than point *SS*), showing Gaussian-like distribution with 2.5% variation as measured from the full-width at half-maximum. Sub-60 mV/dec switching was observed in 68% (34 out of 50) devices.

To demonstrate the advantage of sub-thermionic OTFTs, we fabricated monolayer OTFTs on 24 nm Al_2_O_3_/Si substrate (without HZO). The minimum *SS* of these “normal” devices was 65–75 mV/dec and degraded quickly with *I*_ds_ (Supplementary Fig. 8a). Consequently, sub-thermionic OTFTs delivered more than one order of magnitude higher on-current and *g*_m_ under the same *V*_dd_ (Supplementary Fig. 8). These observations were consistent with a recent theoretical model based on realistic capacitance analysis^23^.

To gain further insight of the device physics, we performed quantitative modeling using an equivalent circuit model in Supplementary Fig. 9. To achieve simultaneous hysteresis-free and sub-thermionic switching, the ferroelectric HZO capacitance (*C*_FE_) needs to fall between that of Al_2_O_3_ (*C*_AlOx_) and $C_{{\mathrm{MOS}}} \approx \big( {C_{{\mathrm{AlO}}_{\mathrm{x}}}^{ - 1} \,+\, \big( {C_{{\mathrm{j}},{\mathrm{S}}} \,+\, C_{{\mathrm{j}},{\mathrm{D}}}} \big)^{ - 1}} \big)^{ - 1}$, where *C*_j,S/D_ are S/D Schottky junction capacitances (Supplementary Fig. 9)^23^. The low dielectric constant and low channel doping of organic channel generate *C*_j,S/D_ ≈ 6 × 10^−7^ F/cm^2^, which is about one order of magnitude smaller than *C*_AlOx_ (3.6 × 10^−6^ F/cm^2^, 2 nm Al_2_O_3_). This defines an extended design space for *C*_FE_. The thin Al_2_O_3_ also reduces the minimum thickness of HZO to achieve sub-60 mV/dec switching. Supplementary Figure 9d (solid blue line) plots *SS* as a function of $C_{FE}^{ - 1}$ using our experimental device geometry. The *C*_FE_ of 22 nm HZO (derived from fitting of P-E loop, Supplementary Fig. 1d) gives *SS* = 56.5 mV/dec (red star), in good agreement with experimental data. The correct capacitance matching of sub-thermionic OTFTs also makes the hysteresis of *I*_ds_ − *V*_gs_ curve negligible^16,23^. Furthermore, we quantitatively calculated the transistor characteristics using a compact model incorporating time-dependent depolarization and multi-domain Landau–Khalatnikov theory^37^. Supplementary Figure 9e, f shows that the calculated transfer and output curves match experiments very well, which validates the above model analysis.

We further benchmark our sub-thermionic OTFTs with other TFT technologies. Figure 2g compares the *g*_m_ and on/off ratio (data from ten devices) with several representative OTFT technologies, under the same |*V*_gs(on)_ − *V*_gs(off)_| = 1 V (see Supplementary Table 2 for details). Our data points are located at the upper right corner of the plot, exhibiting significantly improved *g*_m_ while maintaining on/off ratio greater than 10^8^. Figure 2h summarizes the intrinsic gain of various TFT technologies. Our monolayer sub-thermionic OTFTs exhibit the highest *A*_i_ of 5.3 × 10^4^, which is more than one order of magnitude higher than oxides^38^ and Schottky barrier TFTs^4,39^, and more than two orders of magnitude higher than 2D materials^40^ and silicon. Other than *g*_m_, an important reason for the high *A*_i_ is large *r*_0_ ~10^10^ Ω due to near-zero DIBL in the subthreshold region (Supplementary Fig. 10). This can be understood as a consequence of the transition from negative to positive DIBL, leading to infinite *r*_0_ at the transition (Supplementary Fig. 10). This is a unique property of NC transistors^16,29^ and is also captured by our modeling (Supplementary Fig. 9g). It is important to note that the overall improvement of transistor performance (*g*_m_, *r*_0_, *A*_i_, *SS*) is accomplished by systematic optimization, including high mobility material, monolayer channel, clean contact interface and ferroelectric dielectrics. To verify the importance of monolayer channel, we fabricated devices with thicker channel and observed inferior *SS*, on-current and *g*_m_ due to poor electrostatics and contact resistance^35,41^ (Supplementary Fig. 8).

The devices also had good temperature and operational stability. The sub-60 mV/dec switching was preserved after 2-month storage at room temperature, with only slight shift of *V*_th_ and decrease of on-current (Supplementary Fig. 11a). Variable-temperature measurements showed that the *SS* was below thermionic limit from 250 to 375 K (Supplementary Fig. 11e, f), owing to the high Curie temperature of the HZO^19^. In addition, continuous bias stress measurements under *V*_ds_ = *V*_gs_ = −1.5 V also showed little degradation of device performance after 10^4^ s (Supplementary Fig. 11b, c). Further, we conducted cycle stability test for the transistors, showing excellent cycle stability under repeated gate voltage pulses with 1 Hz frequency (Supplementary Fig. 11d). The stability can be further improved by proper passivation. In terms of frequency limit, previous studies have shown that HZO can properly function up to MHz^42^, which is adequate for OTFT applications.

### Ultra-high-gain amplifier circuits

**Fig. 3:**
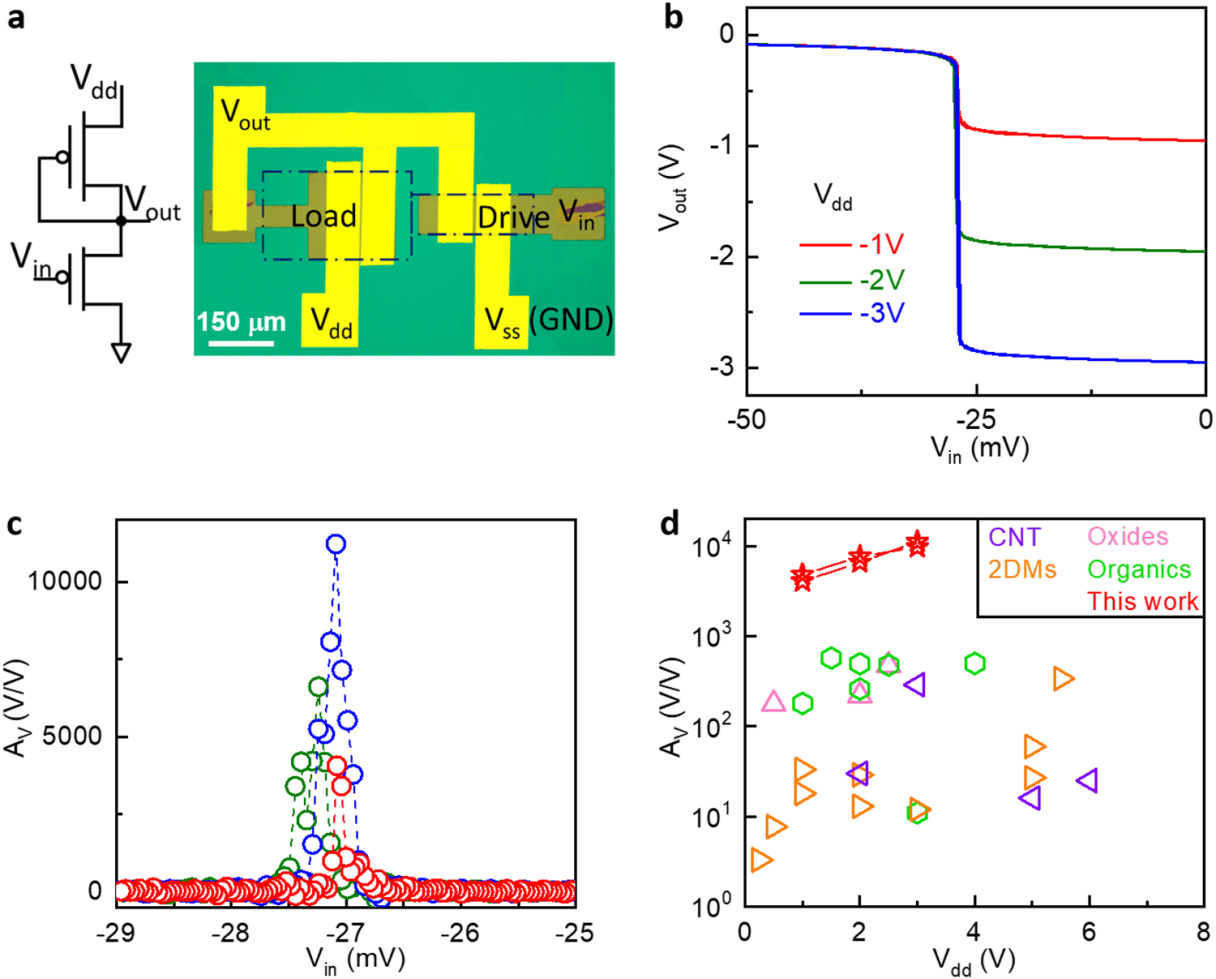
Integrated amplifier based on sub-thermionic monolayer OTFTs. **a** Equivalent circuit diagram (left) and optical microscope image (right) of an enhancement-depletion mode amplifier. The dotted boxes indicate organic film areas for load and drive transistors. **b** Voltage transfer characteristics under *V*_dd_ = −1, −2 and −3 V, respectively. **c** *A*_v_ as a function of input voltage derived from **b**. **d** Comparison of *A*_v_ among similar amplifier circuits using different materials and processes. The extracted data are listed in Supplementary Table 3.

By designing proper electrode patterns, we could build functional circuits, such as inverter and logic gates using local backgate devices (Fig. 3, Supplementary Fig. 12, see “Methods” section for detailed fabrication processes). There is practically no limit in the circuit complexity by vdW integration as long as the transistors are co-planar. For very large-scale integration that requires multiple layers of interconnects, the upper layers can be fabricated by conventional lithography or printing as they are separated from the channel. Figure 3a shows the circuit and optical image of an enhancement-depletion mode inverter using two sub-thermionic OTFTs with different dimensions (the transfer characteristics are presented in Supplementary Fig. 13a). The inverter exhibited full swing output near zero input voltage with peak power of ~50 nW (Fig. 3b and Supplementary Fig. 13b). Remarkably, we obtained giant voltage gain (*A*_v_) of 4.1 × 10^3^ (1.1 × 10^4^) under *V*_dd_ = −1 V (−3 V) (Fig. 3c). On the other hand, control amplifiers fabricated on Al_2_O_3_/Si substrate showed 40 times lower *A*_v_ (Supplementary Fig. 13c, d). Therefore, we conclude that the high gain is enabled by steep switching in the transistor characteristics, which are unique properties of sub-thermionic OTFTs. Figure 3d benchmarks the *A*_v_ of similar amplifier structures based on organics, metal oxides, 2D materials and carbon nanotubes (see Supplementary Table 3 for details). The gain of our devices again outperforms these existing technologies by orders of magnitude while maintaining low *V*_dd_, which hold promise for wearable electronics and IoT applications.

### Flexible sub-thermionic OTFTs and circuits

**Fig. 4:**
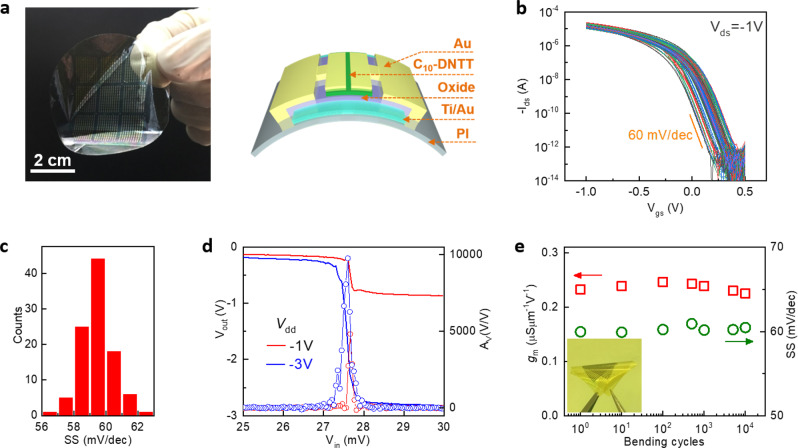
Sub-thermionic OTFTs on flexible substrates. **a** Photograph of OTFT arrays fabricated on 2-in. polyimide substrate (left) and schematic view of the device structure (right). **b** *I*_ds_ − *V*_gs_ curves of 100 devices under *V*_ds_ = −1 V. The *L*/*W* of device channel is 5 μm/180 μm. **c** Statistical distributions of *SS* for the devices in **b**. **d** Voltage transfer characteristics (solid lines) and *A*_v_ (symbols) of an enhancement-depletion mode amplifier on flexible substrate, under *V*_dd_ = −1 and −3 V. **e** Bending test of a device on polyimide substrate. Both *g*_m_ and *SS* remain stable during 10^4^ bending cycles. Inset shows a photograph of flexible device arrays under bending.

For wearable applications, it is important to examine the device performance on flexible substrates. To this end, polyimide was used because it was compatible with the processing temperature of HZO. Spin-coated polyimide (~10 μm thick) on sacrificial Si substrate ensured low surface roughness for solution shearing and mechanical reliability under extreme folding or crumpling. Figure 4a illustrates the structure and photograph of device arrays with local backgates. The transfer characteristics of 100 devices exhibit small variations in *V*_th_ and *SS* (Fig. 4b, c). Importantly, the steep slope and high gain were preserved on polyimide substrate, with 75% (75 out of 100) devices exhibiting sub-60 mV/dec switching and lowest *SS* = 56.5 mV/dec (Fig. 4c). Figure 4d presents the transfer curves of a flexible inverter with *A*_v_ = 9.7 × 10^3^, which was comparable to that on Si substrate. Furthermore, the devices were robust under mechanical bending. Both *g*_m_ and *SS* showed little degradation during 10^4^ repeated bending tests to 10 mm radius of curvature (Fig. 4e).

### Integrated wearable ECG and pulse sensors

**Fig. 5:**
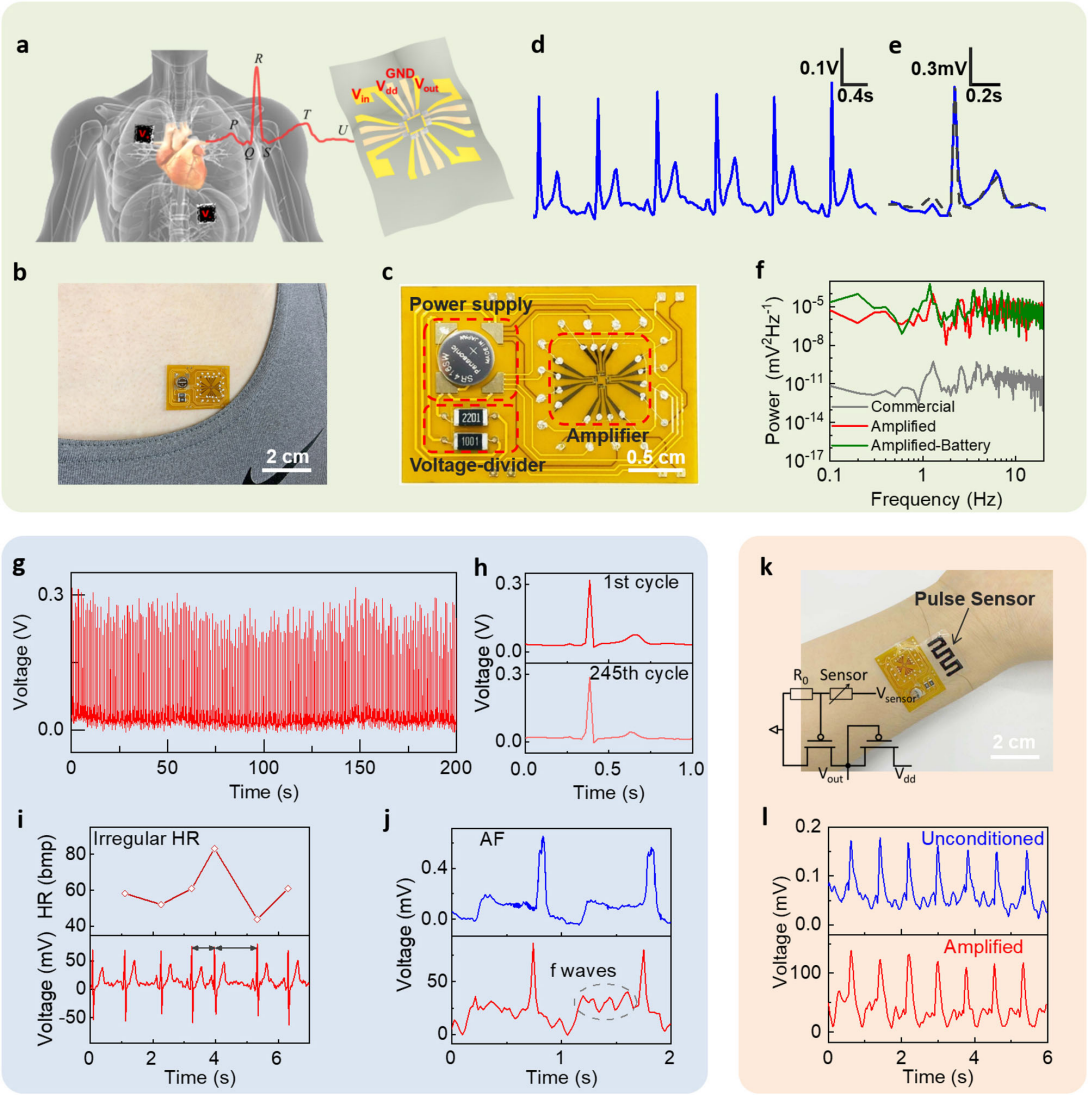
Low-voltage- and high-gain-integrated wearable sensors. **a** Cartoon illustration of the use of amplifier circuit to acquire human ECG signals. **b** Photograph of a flexible amplifier module attached to a human chest. **c** Photograph of the amplifier module, with a coin battery, a voltage divider and amplifier circuit integrated on a flexible circuit board. **d** Amplified ECG signal of a human subject. **e** Deconvoluted ECG signal using the amplifier’s transfer curve (blue solid line) and the ECG signal taken by commercial equipment on the same human subject (black-dashed line). **f** The power spectral density of ECG signals taken by commercial equipment (gray), our amplifier with external voltage source (red) and battery (green) as power supply. **g** Long-term ECG monitoring using our sensor. **h** The ECG signal of the 1st (upper panel) and 245th (lower panel) cycle in **g**. **i** Amplified ECG signal of a human subject with known arrhythmia history (bottom panel) and the corresponding heart rate (upper panel). **j** ECG signal of another human subject with AF, recorded by our sensor (bottom panel) and a clinical equipment (upper panel). **k** Photograph of an integrated pulse sensor using flexible carbon nanotube films and our amplifier circuit. Inset shows the circuit diagram. **l** Unconditioned and amplified pulse signal taken on the same human subject.

Next, we demonstrated the applications of sub-thermionic OTFT technology in wearable electrocardiogram (ECG) and pulse sensors. Human body constantly produces vital physiological signals, such as biopotentials and metabolites, in the range of microvolt to millivolt^43–46^. Therefore, on-site signal amplification using low-voltage and high-gain circuitry is essential for integrated wearable sensors but very challenging. We integrated a flexible enhancement-depletion mode amplifier on a custom-designed flexible circuit board, which could be easily laminated on the front chest (Fig. 5a, b). All the amplifiers were operated under *V*_dd_ = −1 V with the peak *A*_v_ greater than 1000. The *V*_dd_ was supplied either by an external voltage source or a 1.5 V coin battery (Fig. 5c, a voltage divider was used to reduce *V*_dd_), showing similar performance (Supplementary Fig. 14). Figure 5d plots the amplified ECG signal of a healthy male subject, clearly showing all the expected waveforms (P, Q, R, S, T and U waves). The signal amplitude was ~350 mV, corresponding to an amplification factor of 324. The raw ECG signal could be obtained by deconvoluting the amplifier transfer curve (the blue solid line in Fig. 5e, see Supplementary Fig. 15 for details), which agreed well with the data recorded by a clinical grade commercial equipment (Prince 180B by Heal Force, the gray-dashed line in Fig. 5e). Figure 5g, h demonstrates continuous monitoring of ECG over several minutes. The signal fidelity, waveform and amplification factor remained stable during the test, which was essential for long-term health monitoring. To further prove the signal fidelity, we simultaneously measured the ECG signal by our sensor and commercial equipment (Supplementary Movie 1). Figure 5f compares the power spectral density. We can see that the amplified ECG shares the same waveform with the commercial equipment, with the most prominent peak at 1.29 Hz corresponding to the heart rate of the subject. Importantly, the amplitude of power density was enhanced by ~10^5^ times, consistent with the amplification factor of ~300. Battery-powered ECG also showed similar power spectral density.

With excellent sensitivity and signal fidelity, our ECG sensors are capable of facilitating cardiopathy diagnoses. To this end, two hospital volunteers were tested. On subject A (female, mid-20s) who was diagnosed with arrhythmia, our sensor clearly captured the irregular heartbeat with a sudden change from 83 to 43 beats per minute (bpm) (Fig. 5i). On subject B (male, 80 s) who was diagnosed with atrial fibrillation (AF), our sensor was capable of detecting f wave with a frequency of 357 bpm, which was a direct signature of AF (Fig. 5j, lower panel). In contrast, the clinical ECG of same subject did not resolve f wave due to its weak amplitude (Fig. 5j, upper panel, taken by NIHON KOHDEN ECG-1350P). Currently, AF is the most common arrhythmia globally associated with the increased risk of stroke and heart failure. The ability to directly identify f wave will significantly increase the sensitivity and specificity of clinical diagnosis. Without any post-processing, the signal-to-noise ratio (SNR) of our ECG signal reached 42 dB, exceeding previously reported results^47,48^. The improved sensitivity and the ability to detect extremely weak signals are remarkable considering the simple circuity of our sensors.

As another example, we connected the amplifier to a flexible carbon nanotube sensor to measure wrist pulse signals (Fig. 5k). The pulse pressure-induced resistance change in the carbon nanotube film, resulting in a slight shift of amplifier input voltage. Figure 5l shows the measured pulse waveforms of a human subject with ~120 mV peak-to-valley amplitude, corresponding to amplification factor of 900. The details of the pulse waveforms including a systolic peak, a dicrotic notch, a reflected systolic peak and an end-diastolic notch were clearly resolved. The higher amplification compared to ECG was due to the smaller input signal amplitude (~0.13 mV vs. ~1 mV for ECG), which allowed the amplifier to work at the peak gain point. Compared to literatures, our ECG and pulse sensors show advantages in terms of *V*_dd_, gain and SNR (Supplementary Table 4).

## Discussion

We demonstrate the first sub-thermionic flexible OTFT technology by combining solution-processed monolayer organic channel, ferroelectric gate dielectrics and vdW device integration. The devices operate under one-volt and exhibit sub-60 mV/dec switching and record-high intrinsic gain. We further build amplifier circuits with ultra-high voltage gain of 1.1 × 10^4^, which outperform existing TFT technologies by more than one order of magnitude. The flexible amplifiers are capable of amplifying human physiological signals by several hundred times with high fidelity, and detecting extremely weak ECG signals to facilitate clinical diagnosis. By developing more robust amplification schemes (such as differential amplification)^48^ and wireless data transmission^49^, we can envision all-organic, battery-powered patches for high-precision health monitoring and real-time cloud-based diagnosis.

## Methods

### Preparation of HZO/Al_2_O_3_ dielectrics

The HZO film was deposited by ALD on p++ silicon (100) substrate at 200 °C and base pressure of ~1 Pa using Tetrakis(dimethylamido) hafnium as Hf source, Tetrakis(dimethylamido) zirconium as Zr source and H_2_O as oxidant. We used 20 sccm N_2_ as carrier gas. The HfO_x_ and ZrO_x_ were deposited sequentially with 1:1 ratio. Then 2 nm AlO_x_ capping layer was deposited by ALD in situ for capacitance matching, using trimethylaluminium and H_2_O as sources. The pulse/purge time for Hf, Zr and H_2_O precursors were 250 ms/30 s, 20 ms/30 s and 30 ms/30 s, respectively. The substrate was undergone 350 °C rapid thermal annealing for 1 min in N_2_ to crystallize the HZO.

We fabricated the HZO/Al_2_O_3_ dielectrics on polyimide substrate using the similar method mentioned above. To prepare the polyimide substrate, we spin-coated polyimide solution (AA-49, KANEKA) on SiO_2_/Si substrate, and then baked at 350 °C for 1 h on a hotplate.

### Solution shearing of monolayer C_10_-DNTT films

The C_10_-DNTT powder was dissolved in 1,2,3,4-tetrahydronaphthalene (tetralin) at 85 °C with a concentration of 0.2 mg/mL. The growth of monolayer C_10_-DNTT was carried out in a home-built solution shearing setup with heaters on both the blade and substrate (Supplementary Fig. 2). The temperature of the blade and substrate was set to 65 °C. Overall, 150 μL of C_10_-DNTT solution was injected for a 2 in. size substrate and a constant shear velocity of 3 μm/s was established by using a linear translation stage during the shearing processes.

### Characterizations of C_10_-DNTT film and OTFTs

We used ellipsometry mapping, high-resolution AFM and cross-polarized optical microscopy to characterize the monolayer C_10_-DNTT film. The ellipsometry mapping was performed on a Woollam RC2-XI mapping ellipsometer in the wavelength range of 210–2500 nm. For high-resolution AFM, the experiments were conducted on Asylum Cypher under ambient conditions. The cross-polarized optical reflection micrographs were performed by the ScanPro spectro-microscope under the white light. We used cross-sectional TEM to characterize the interface and thickness of sub-thermionic OTFTs. The TEM specimens were prepared using a FEI Scois Dualbeam focused ion beam. After that, the specimens were characterized using a high-resolution JEOL JEM-2100F TEM with acceleration voltage of 200 kV equipped with energy-dispersive spectroscopy detector.

### vdW integration of contacts

First, we prepare patterned gold electrodes with 50 nm thickness on a silicon substrate using EBL and electron-beam evaporation (EBE). Next, a PMMA layer was spin-coated, followed by the lamination of thermal released tape (TRT). The TRT/PMMA/Au electrode stack was carefully released from silicon substrate and was align transferred to patterned monolayer C_10_-DNTT film. Then the TRT was separated at 90 °C. Finally, the PMMA layer was patterned by EBL to remove the PMMA on probing pads for electrical measurements.

### Fabrication of amplifiers with local backgate

First, we fabricated 10 nm Ti/20 nm Au backgate electrodes on SiO_2_/Si substrate using EBL and EBE. Then, HZO/Al_2_O_3_ was deposited using ALD. EBL and inductive coupled plasma reactive ion etching (BCl_3_ and Cl_2_) were adopted to etch HZO/Al_2_O_3_ and open via holes to backgate electrodes. Then, monolayer C_10_-DNTT films were grown by the solution shearing process. Finally, the source/drain electrodes (50 nm Au) were align transferred onto the C_10_-DNTT films. For the device on the polyimide substrate, the processes are similar.

### Electrical measurements

Electrical measurements were carried out by an Agilent B1500 semiconductor parameter analyzer in a probe station under ambient conditions. And the temperature during the OTFT’s electrical measurement was set at 300 K.

### Electrocardiogram and pulse detection

The flexible amplifier connected with two conventional gel electrodes to obtain the ECG signal. One was placed on the skin above the lower left part of heart and connected to a bias voltage to set the signal at the amplifier optimal work point. And another was placed on the upper right part of the heart as the input signal of the amplifier. We use Agilent B1500 to collect ECG signal and the sampling frequency was 40 Hz. Fast Fourier transforms were applied to identify the power density of the recorded ECG signal. The SNR was calculated by the maximum peak value of the signal divided by the root mean square of the noise area. Inset of Fig. 5k shows the circuit diagram for pulse detection. The resistance of the flexible carbon nanotube sensor is about 1 kΩ. By connecting it with a 1 kΩ divider resistor, the resistance change of the sensor was converted into a voltage signal, then as the input of the amplifier. Agilent B1500 was used to collect the pulse signal.

The authors affirm that human research participants provided informed consent for publication of the video in Supplementary Movie 1. All human subjects involved in the ECG and pulse tests provided informed consent, and the study protocol was approved by local ethics committee on human research of Nanjing Drum Tower Hospital (2019-190-01).

## Supplementary information

Supplementary Information

Supplementary Movie 1

Description of Additional Supplementary Files

## Data Availability

All data that support the findings of this study are available from the corresponding author upon reasonable request.
