# Supplementary material for: Sub-thermionic, ultra-high-gain organic transistors and circuits
Source: Nat Commun. 2021 Mar 26;12:1928. doi: 10.1038/s41467-021-22192-2 (PMC7997979; doi:10.1038/s41467-021-22192-2)
Supplement: Supplementary file 1 — Supplementary Information [file 41467_2021_22192_MOESM1_ESM.pdf]

Supplementary Information for

Sub-thermionic, ultra-high-gain organic transistors and circuits

Zhongzhong Luo[1]†, Boyu Peng[2,3]†, Junpeng Zeng[1]†, Zhihao Yu[1,4]†, Ying Zhao[5], Jun Xie[6], Rongfang Lan[6], Zhong Ma[1], Lijia Pan[1], Ke Cao[7], Yang Lu[7], Daowei He[1], Hongkai Ning[1], Wanqing Meng[1], Yang Yang[1], Xiaoqing Chen[1], Weisheng Li[1], Jiawei Wang[5], Danfeng Pan[1,8], Xuecou Tu[1,8], Wenxing Huo[9], Xian Huang[9], Dongquan Shi[10], Ling Li[5], Ming Liu[5], Yi Shi[1], Xue Feng[11], Paddy K. L. Chan[2,12*], Xinran Wang[1*]

*Correspondence to: xrwang@nju.edu.cn, pklc@hku.hk

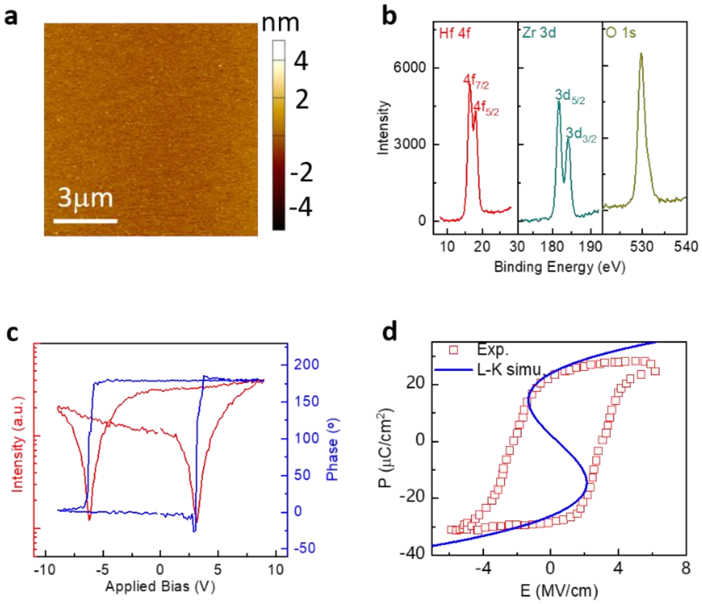

Supplementary Figure 1| Characterization of HZO dielectric. a, AFM image and **b,** XPS of the HZO film on Si substrate. The composition of Hf:Zr = 1:0.95. **c,** Single-point PFM characterization showing ferroelectric behavior of HZO. **d,** The experiment Polarization-electric field loop of Si/ 22 nm HZO/Au stack showing hysteretic behavior (red square). The blue line is the fitting curve using the L-K equation to extract the Landau coefficients.

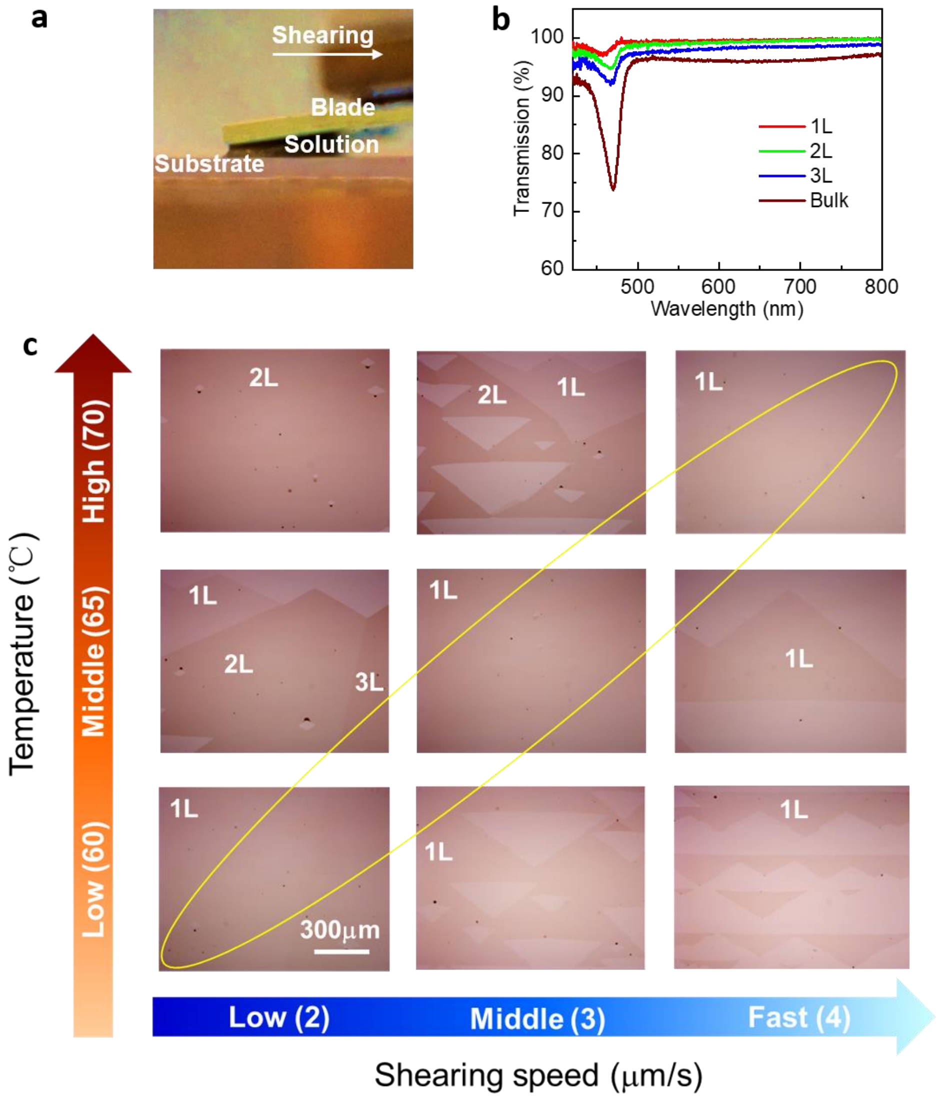

Supplementary Figure 2| Solution shearing of C_{10}-DNTT films. a, Photograph of the shearing process. **b,** Transmission spectra of C_{10}-DNTT films at different number of layers. **c,** The film morphology depends on the substrate temperature and shearing speed. By optimizing the growth condition, uniform and large area monolayer could be realized.

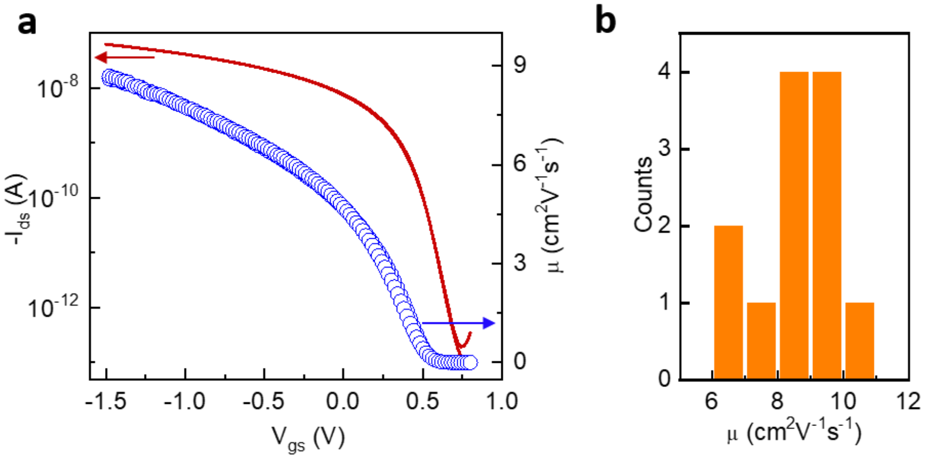

Supplementary Figure 3| Mobility of monolayer OTFTs on Al_2O_3 dielectrics. a, I_{ds}-V_{gs} characteristics (V_{ds} = -0.1 V, red line) and the extracted field-effect mobility as a function of V_{gs} (blue symbols) of a monolayer OTFT on Al2O3 dielectric. b, Statistical distributions of mobility for 12 devices. The length to width ratio (L/W) of device channel is 150 μm/50 μm.

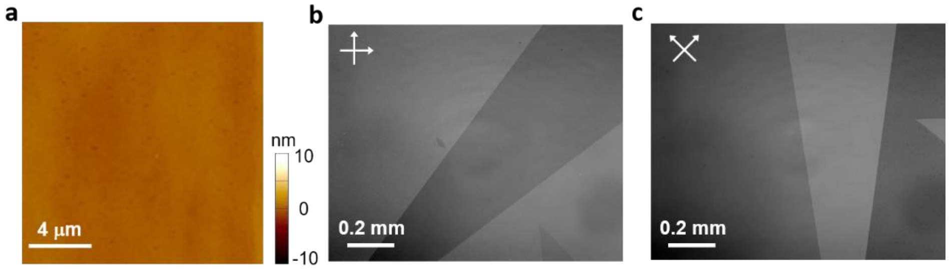

Supplementary Figure 4| Characteristics for monolayer C_{10}-DNTT film on flexible substrate. a, AFM and **b,c,** Cross-polarized optical micrograph of monolayer C_{10}-DNTT film on polyimide substrate.

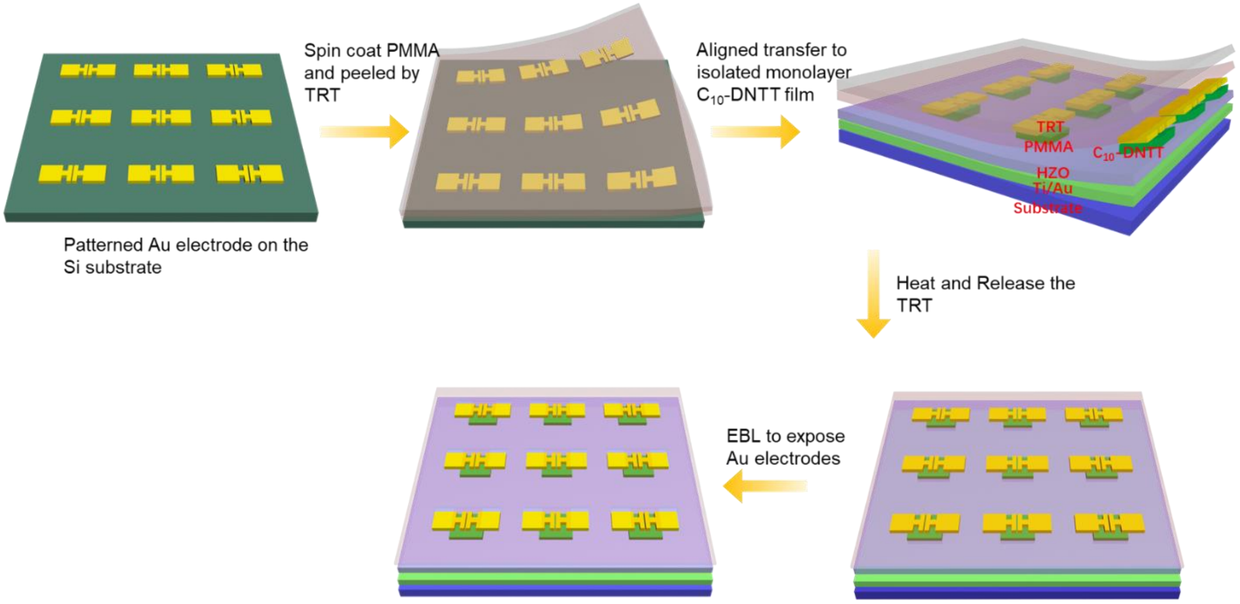

Supplementary Figure 5| Schematic illustration of vdW fabrication process for sub-thermionic OTFTs array.

1) Fabricate the patterned Au electrode on the Si substrate using EBL and EBE;

2) Spin coat PMMA and then peel the electrode and PMMA using thermal released tape (TRT);

3) Aligned transfer to patterned monolayer C_{10}-DNTT film;

4) Heat at 90 °C to release the TRT;

5) Perform EBL to pattern the PMMA to expose Au electrodes;

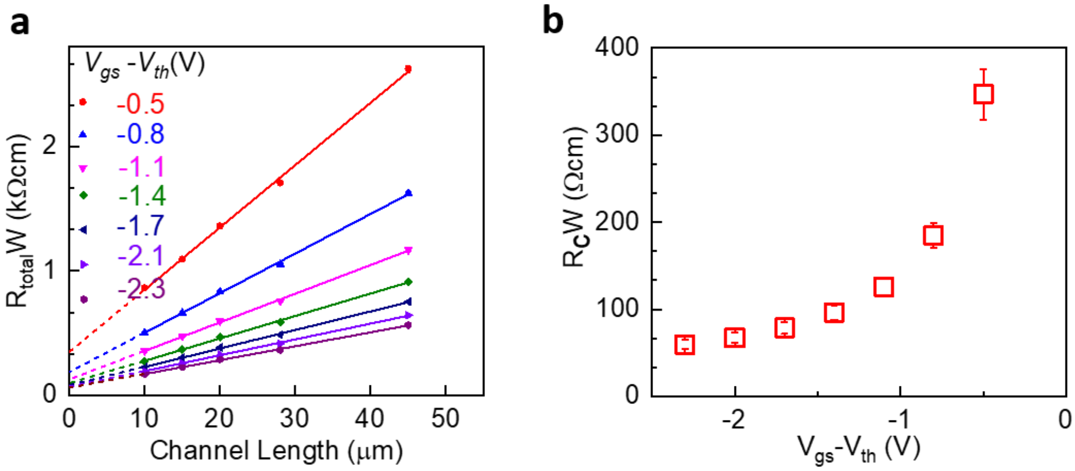

Supplementary Figure 6| Contact resistance measurement. a, Linear fits to the total width-normalized resistance ($R_{total}W$) at different gate overdrive voltages. **b,** Width-normalized contact resistance ($R_C W$) plotted as a function of the gate-overdrive voltage. Error bars are derived from linear fitting errors.

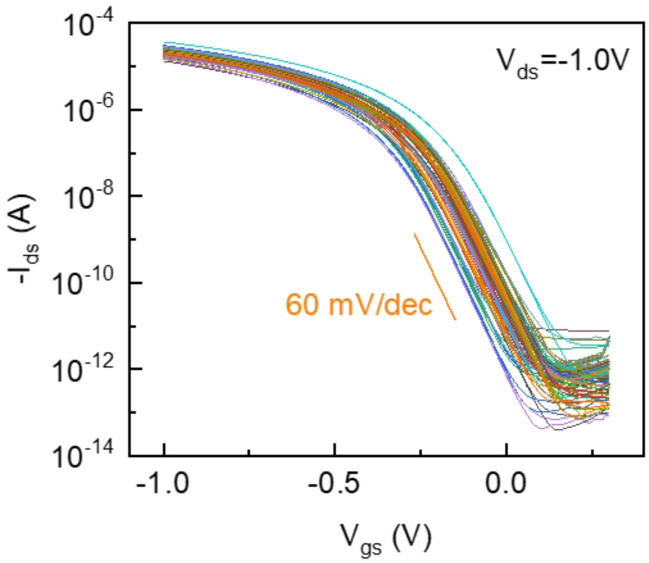

Supplementary Figure 7| Transfer characteristics of 50 sub-thermionic monolayer OTFTs.

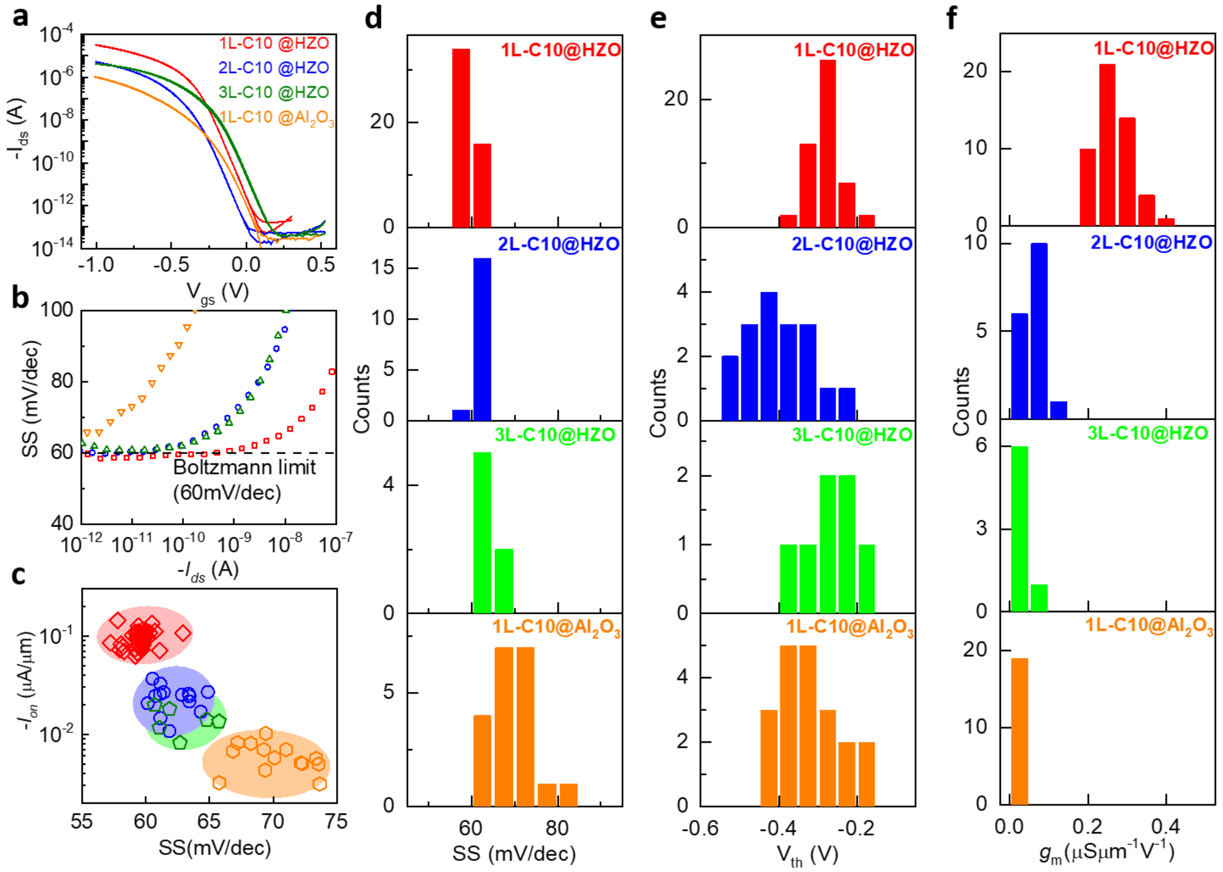

Supplementary Figure 8| Thickness and substrate dependence of the OTFTs. a, I_{ds}-V_{gs} characteristics of typical monolayer, bi-layer and tri-layer C_{10}-DNTT OTFTs on 22 nm HZO/2 nm Al_2O_3 as well as monolayer C_{10}-DNTT OTFT on 24 nm Al_2O_3 under V_{ds} = -1 V. **b,** *SS* versus I_{ds} characteristics of the devices in **a**. **c,** Statistical analysis of I_{on} vs *SS* for monolayer (red), bi-layer (blue) and tri-layer (green) C_{10}-DNTT OTFTs on 22 nm HZO/ 2nm Al_2O_3 as well as monolayer C_{10}-DNTT OTFTs on 24 nm Al_2O_3 (orange). I_{on} is defined as the current at $|V_{ds}|$ = $|V_{gs(on)}$-$V_{gs(off)}|$ = 1 V. Statistical distribution of *SS* (**d**), V_{th} (**e**) and normalized $\boldsymbol{g_m}$ (**f**) for different thickness and substrates.

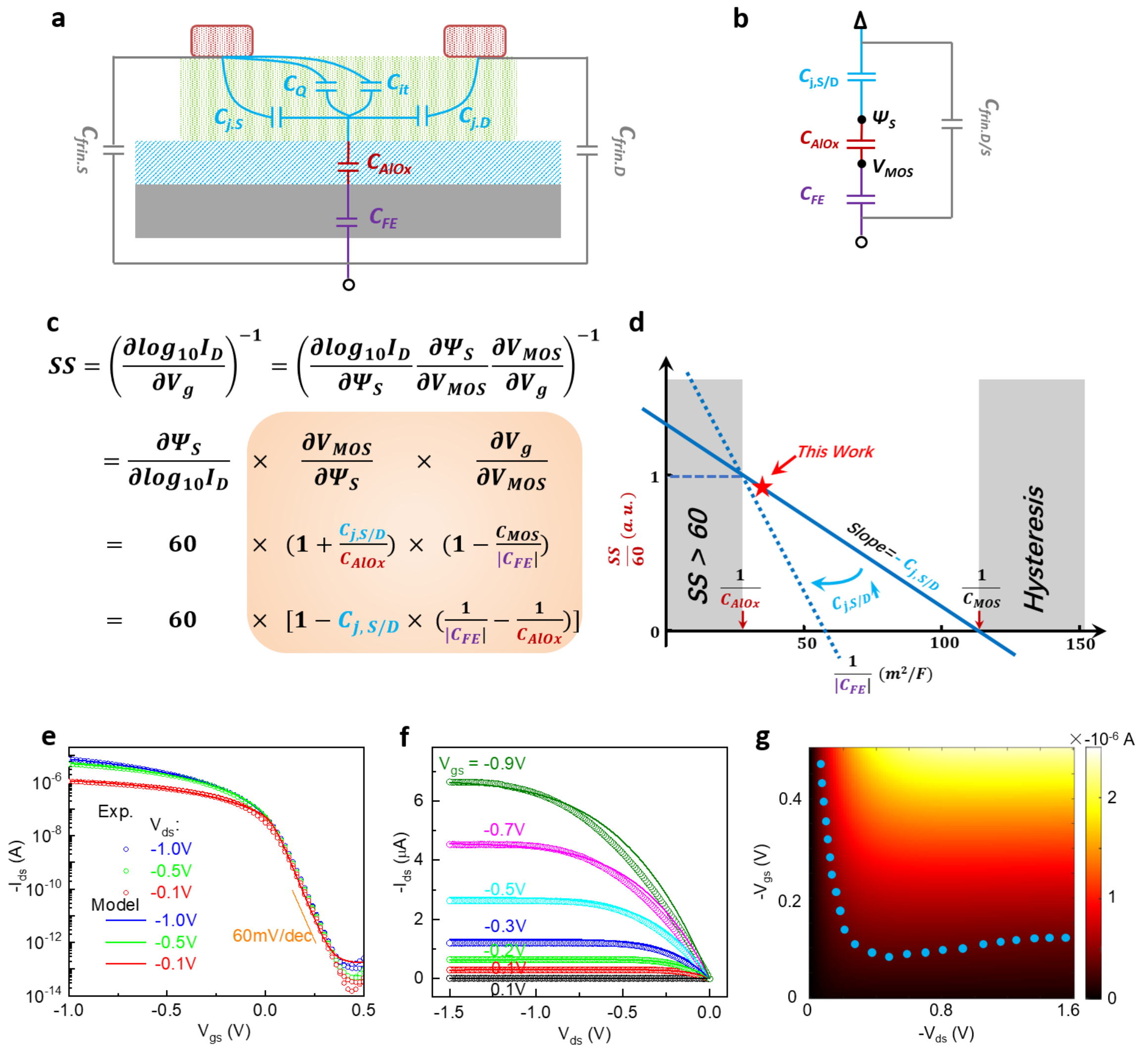

Supplementary Figure 9| Device model of sub-thermionic monolayer OTFT. a, Capacitors network and **b,** simplified small-signal model of sub-thermionic OTFTs. **c,** Formulating *SS* by decoupling the electrostatics and transport contributions. **d,** Graphical display of the dependence of *SS* on capacitance. The region of $\frac{1}{C_{AlOx}} < \frac{1}{|C_{FE}|} < \frac{1}{C_{MOS}}$ is the design space for capacitance matching, where SS < 60 mV/dec without hysteresis. In the left shade region ($\frac{1}{|C_{FE}|} < \frac{1}{C_{AlOx}}$), $SS >$ 60 mv/dec. In the right shade region ($\frac{1}{|C_{FE}|} > \frac{1}{C_{MOS}}$), $SS < 0$, hysteresis appears. The dashed line illustrates the ultra-small window for capacitance matching, when the $C_{j,S/D}$ increases. **e,f,** Experimental transfer and output characteristics (symbols) and the model fitting result (solid line). The model successfully reproduces the experimental data. **g,** Calculated output curves at

different V_{gs} and the blue points are I_{ds} = -0.5 μA, showing negative to positive differential resistance crossover, which is responsible for the very large r_0.

The ferroelectric (FE) properties of HZO can be described as double-well energy landscape by the Landau Khalatnikov (L-K) equation. According to the L-K equation, in the interval between coercive fields, the value of differential capacitance C_{FE} is the negative, which is also metastable state regime. Through proper capacitance matching, we can stabilize the metastable negative capacitance (NC) range, and the NC effect can be used to realize sub-thermionic transport. The Landau coefficients can be extracted by fitting the experimental P-E curve of HZO ferroelectric capacitor to calculate the ferroelectric capacitance C_{FE}. Figure. S1d shows the best fitting results through L-K equation with $\alpha = -7.7 \times 10^8$ m/F and $\beta = 2.8 \times 10^9$ m^5/(F coul^2), and the corresponding $c_{FE} \approx \frac{1}{2\alpha t_{FE}} = -2.8$ μF/cm^2.

We extend the model in Ref. 1 for quantitative analysis and modify a physical based NC-FET analytical model[2] for our 2D organic monolayer structure, incorporating the multi-domain L-K theory with the domain interaction term, polarization relaxation and semi-classical Boltzmann transport theory. To predict the characteristics of the sub-thermionic OTFT and understand more details of the mechanisms, we depict all relevant capacitors in our back-gate sub-thermionic OTFT in Supplementary Fig. 9a. C_{FE} and C_{AlOx} are the capacitance of HZO layer and Al_2O_3 layer, respectively. C_Q, C_{it}, and $C_{j,S/D}$ represent quantum capacitance (negligible in subthreshold regime of OTFT), interface trap induced capacitance (negligible due to the small value), and capacitance of Schottky junction under source/drain electrodes, respectively. Therefore, $C_{j,S/D} = \frac{\varepsilon_O}{t_j}$ (where ε_O is dielectric constant of organic layer, which is about 3[3], and t_j is thickness of Schottky junction[4]) is the dominant capacitance in the channel region. Due to the low doping concentration of the ultra-thin channel, t_j is close to the monolayer molecular thickness of 4 nm, and the corresponding $C_{j,S/D} = 6 \times 10^{-7}$ F/cm^2. In addition, C_{AlOx} is measured to be 3.6×10^{-6} F/cm^2. Supplementary Fig. 9b plots the simplified small-signal model of sub-thermionic OTFTs. It is worth noting that source/drain-to-gate fringing capacitance C_{frin} is connected in parallel outside the capacitor network that modifies channel charges, and does not contribute to capacitor matching. Based on the analytical model in Supplementary Fig. 9c, we can plot the relationship of SS versus $1/|C_{FE}|$ in Supplementary Fig. 9d. To satisfy non-

hysteretic conditions, $1/|C_{FE}|$ need to be smaller than $1/C_{MOS}$ ($1/C_{MOS} = 1/C_{j,S/D} + 1/C_{AlOx}$), while to maintain SS < 60mV/dec, $1/|C_{FE}|$ need to be greater than $1/C_{AlOx}$. $|C_{FE}|$ in the optimal NC-FET should fall between C_{AlOx} and C_{MOS}. Therefore, as shown in Supplementary Fig. 9d, reduced $C_{j,S/D}$ will enlarge the $|C_{FE}|$ design space significantly. Meanwhile, small $C_{j,S/D}$ appears as smaller slope in Supplementary Fig. 9d, which will make it more difficult to reduce SS because extremely thick ferroelectric will be needed.

It is essential to extract the key parameters using capacitor network above in real device after setting up the whole physical model of sub-thermionic OTFT, to guarantee the reasonable values. The surface potential φ_s is the clue of the whole model, obtained by solving the voltage balance condition equation in the vertical direction of sub-thermionic OTFT via the special method [2]. The voltage across the FE layer, V_{FE}, is obtained by L-K theory, as Eq. (1).

$$V_{FE} = (2\alpha Q_G + 4\beta {Q_G}^3 - 4\pi \frac{1}{t_{fe}} \int_0^{t_{fe}} Q_G dy + 4\pi j Q_G - \frac{1}{2} K_P \frac{d^2 Q_G}{dx^2}) t_{fe} \tag{1}$$

The relationship for V_{FE} and P in the Landau theory is a cubic function. α and β can be obtained via the two poles. The third and fourth items in the right part of Eq. (1) represents depolarization field[5] and the last one is multi-domain interaction item[6], both of which modulated severely the negative differential conductance (NDC) effect and well considered as the most possible reason of NC effect. α and β are the Landau coefficients relating to remnant polarization and coercive field. t_{fe} is the thickness of FE layer, and j, K_P are the depolarization and domain interaction coefficients. The gate charge Q_G, which is obtained by the Poisson Equation and modified as areal density for monolayer C_{10}-DNTT, is equivalent to Polarization P when the effect of multi-domain, depolarization has already incorporated as the extra items.

Based on the continuity equation, I-V characteristics can be established. The Current at any position in the channel comprising of drift and diffusion components is given by Eq (2)-Eq (3), with mobility model considering the scattering mechanisms and temperature dependence[7] in Eq (4).

$$I_{drift} = \frac{\mu W}{L} \gamma \frac{2}{3} [\varphi_{ss}^{3/2} - \varphi_{sd}^{3/2}] + \frac{\mu W}{L} \left[\frac{1}{2}(\alpha + 1/C_{AlOx}) {Q_g}^2(\varphi_s) + \frac{3}{4} \beta {Q_g}^4(\varphi_s)\right]_{\varphi_{sd}}^{\varphi_{ss}} \tag{2}$$

$$I_{diffusion} = \frac{\mu W}{L} \varphi_t [Q_i(\varphi_{ss}) - Q_i(\varphi_{sd})] \tag{3}$$

$$\mu = \mu_0 \left\{ \left(1 + U_a E_{eff}^{\frac{1}{3}}\right) \left(\frac{T}{300}\right)^{\frac{3}{2}} + U_b \left(\frac{T}{300}\right)^{-\frac{1}{2}} E_{eff}^2 \left(1 + U_{k_1} V_{bs}\right) \right\}^{-1} \quad (4)$$

The parameter γ including some intrinsic parameters like permittivity, unit charge, body factor. W, L and C_{AlOx} are the width, length of channel and capacitances of Al_2O_3 layer, respectively. φ_t is the thermal voltage and E_{eff} is the effective average transverse electric field in channel, μ_0 is the originate mobility, while U_a, U_b and U_{k_1} are the Semi-fitting parameters representing the strengthen of scattering mechanism.

The current equation can be simplified in linear region, and the originate mobility μ_0 and other parameters like U_b can be extracted from the experimental data. An accurate match between prediction results of the model and experimental data for transfer (in linear and log coordinates) and output characteristics are given in Supplementary Fig. 9e,f, via fine tuning the value of parameters in a reasonable tiny range. Supplementary Fig. 9g plots a mapping diagram of simulated I_{ds} versus V_{ds} and V_{gs}, where the blue points are I_{ds} = -0.5 μA, and the NDC can be clearly captured.

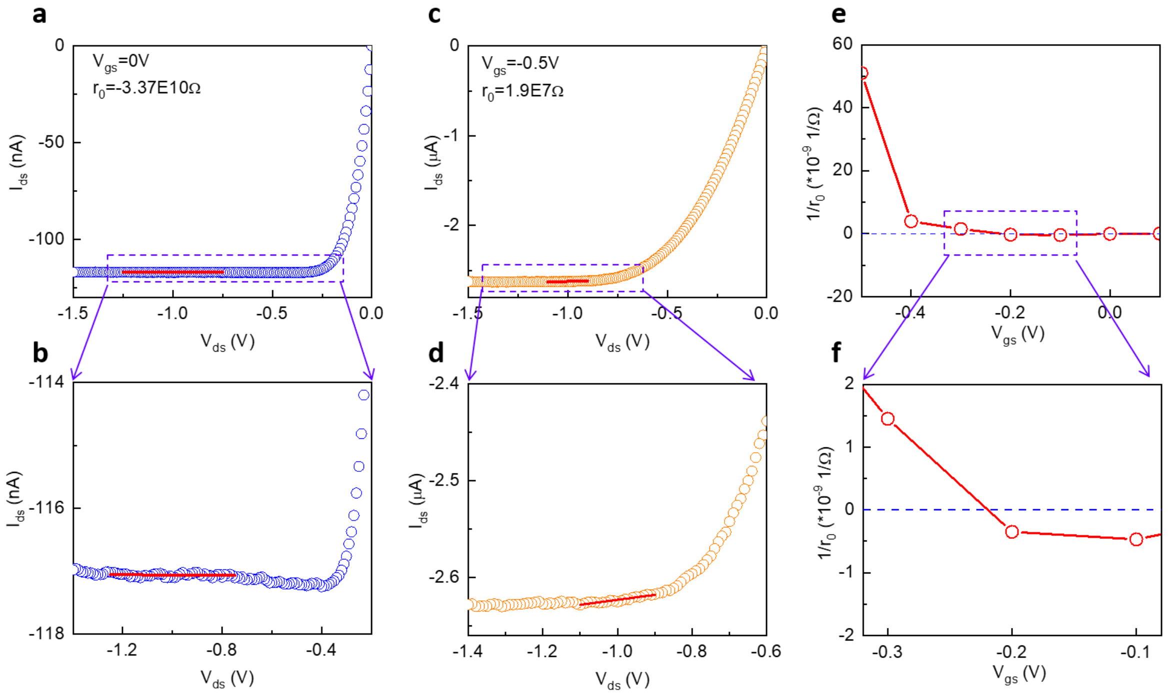

Supplementary Figure 10| Output resistance of the sub-thermionic OTFTs. a-d, Output characteristics at V_{gs} = 0 and -0.5 V of a sub-thermionic OTFT. When V_{gs} = 0 V, the negative differential resistance (NDR) phenomenon appears and the output resistance is negative; when V_{gs} = -0.5 V, the NDR disappears and the output resistance is positive. The red curve is the linear fitting to extract the output resistance. **e,** Output resistance as a function of V_{gs}. **f,** Zoom-in of the curve in **e**, showing a transition of output resistance from negative to positive**.**

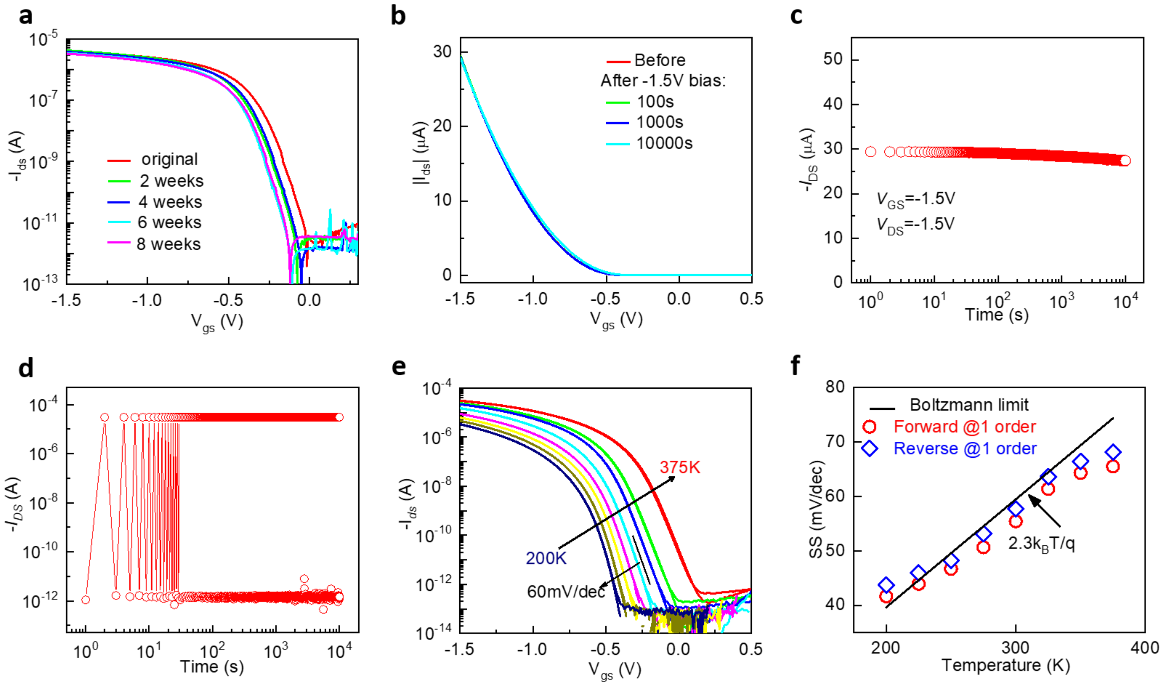

Supplementary Figure 11| Stability of the sub-thermionic OTFTs. a, I_{ds}-V_{gs} characteristics (V_{ds} = -0.1 V) for an OTFT after two-month storage at room-temperature. **b,** I_{ds}-V_{gs} characteristics measured at before and after -1.5 V bias stress test. **c,** Stability of the source-drain current under a continuous bias stress (V_{gs} = -1.5 V, V_{ds} = -1.5 V) over 10000 s. **d,** Cycle stability of the sub-thermionic OTFTs, where a gate voltage pulse (-1.5 V) was applied and the device was switched between on and off for 10000 cycles. **e,** Double-sweep I_{ds}-V_{gs} characteristics (V_{ds} = -1 V) at different temperatures of a sub-thermionic OTFT. **f,** Temperature dependence of *SS* (averaged over one decade of I_{ds}) from 200 to 375 K, showing that the *SS* was below the Boltzmann limit in a wide temperature range. Red, forward sweep; blue, reverse sweep.

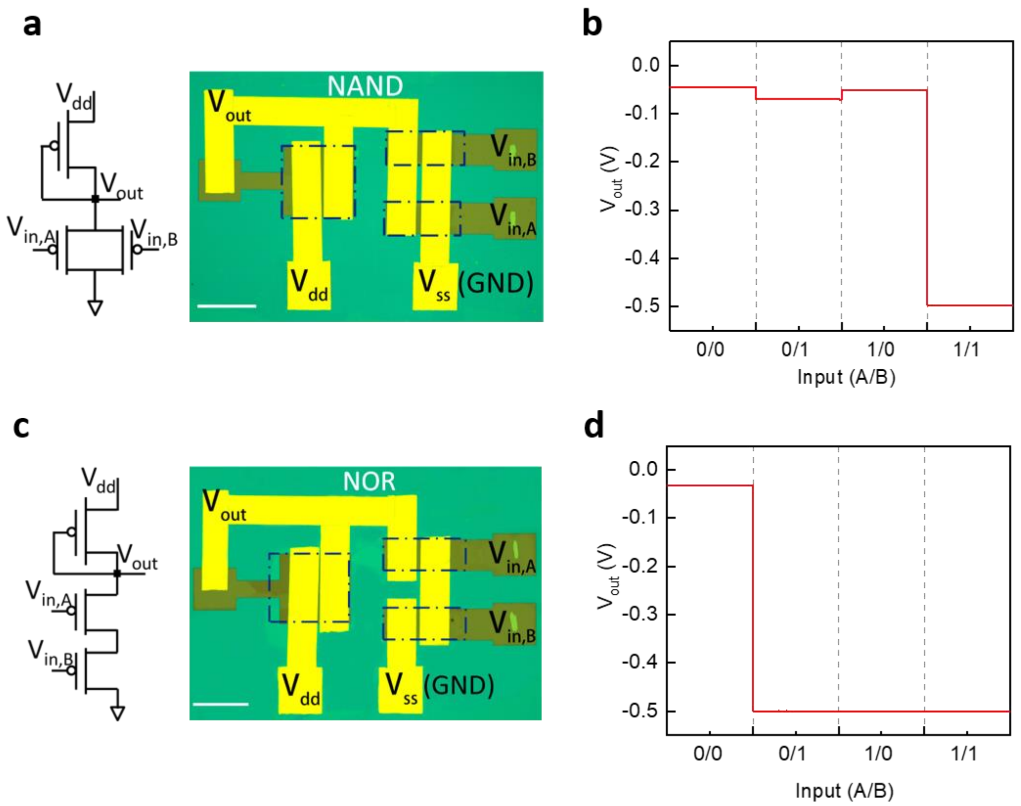

Supplementary Figure 12| Integrated logic gates based on sub-thermionic monolayer OTFT. a,c, Equivalent circuit diagram (left) and optical microscope image (right) of NAND (**a**) and NOR (**c**) gates. The dotted boxes indicate organic film areas for transistors. **b,d,** Output characteristics of NAND (**b**) and NOR (**d**) gates.

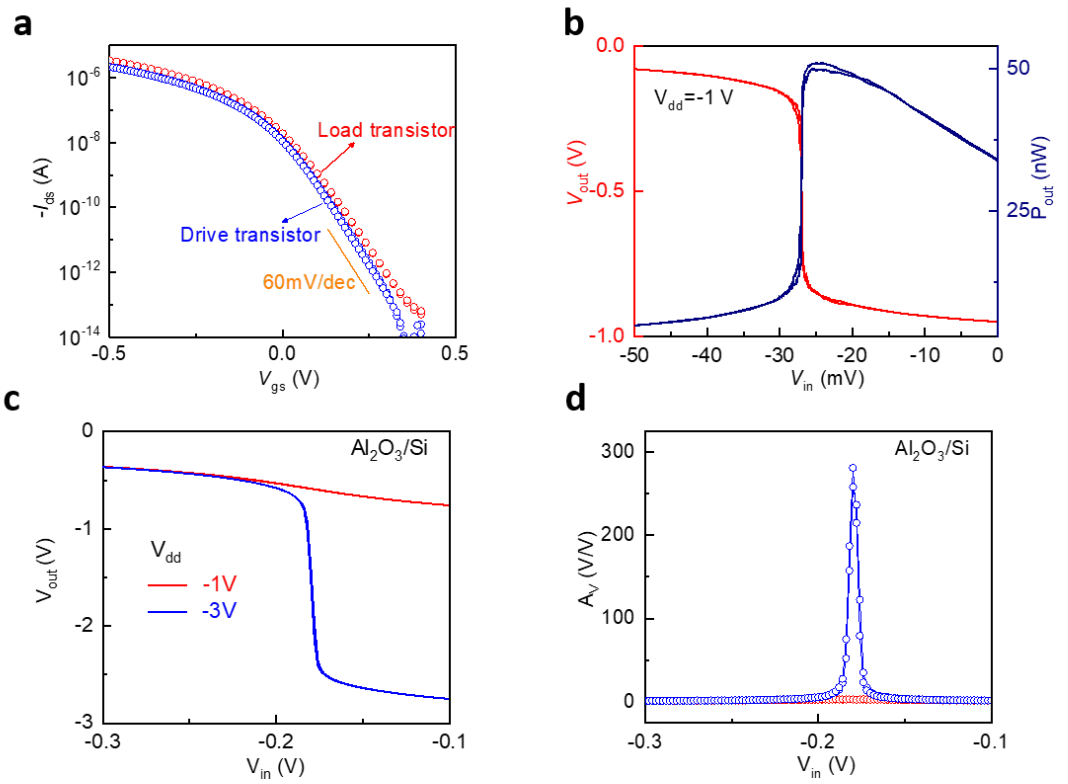

Supplementary Figure 13| Performance comparison of amplifiers on different dielectrics. a, Transfer characteristics of the load (W/L=180/5) and drive (W/L=90/5) transistors. **b,** Measured voltage transfer characteristic and power curve of an inverter on HZO substrate. **c,** Voltage transfer characteristic and **d,** voltage gain of an inverter on Al_2O_3 substrate. At V_{dd} = -1V, the device on normal Al_2O_3 dielectric barely shows any gain, which is dramatically different from sub-thermionic OTFTs.

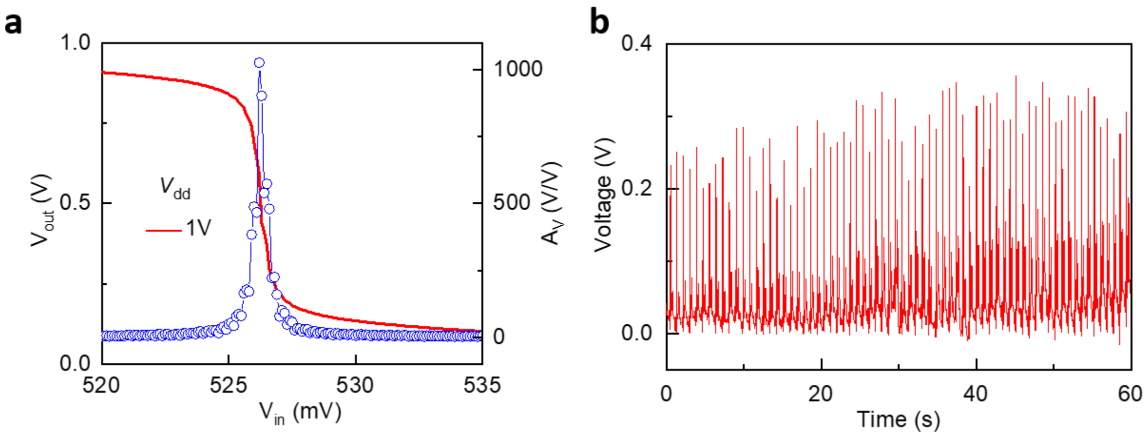

Supplementary Figure 14| Battery-power amplifier module to monitor ECG. a, Voltage transfer characteristic and voltage gain of an inverter powered by a coin battery showing a peak gain greater than 1000 at V_{dd} = 1 V. **b,** ECG monitoring using battery-powered amplifier module.

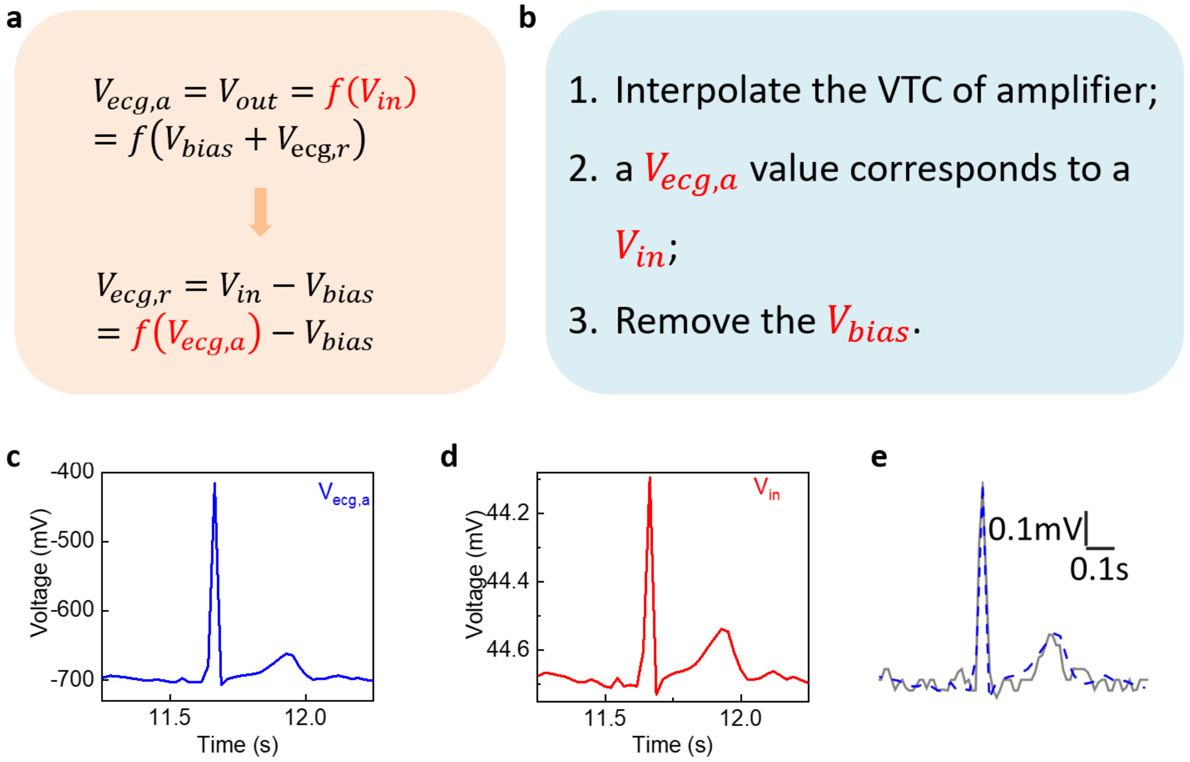

Supplementary Figure 15| ECG deconvolution. a, The principle of ECG signal deconvolution. **b,** The specific deconvolution steps. **c,d,** ECG signal before (**c**) and after (**d**) deconvolution. The gain is calculated to be 459. **e,** Comparison of the deconvoluted ECG signal (blue dashed line) and commercial equipment taken on the same human subject (grey line).

Supplementary Table 1| Contact resistance comparison for OTFTs.

Material	Device structure	Thickness (nm)	Contact technique	R_cW (Ωcm)	Ref.
Rubrene	Bottom-contact	1000	Nickel contact	100	8
Pentacene	Bottom-contact	NA	UV/ozone treatment	80	9
C_8-BTBT	Bottom-contact	40	Doped	100	10
C_8-BTBT	Top-contact	40	Doped	200	10
C_8-BTBT	Top-contact	Monolayer	Graphene contact	100	11
C_8-DNBDT-NW	Top-contact	Bilayer	F_4-TCNQ/Au	46.9	12
DPh-DNTT	Bottom-contact	20	PFBT treatment	29	13
DPh-DNTT	Top-contact	20	Without PFBT treatment	56	13
DPh-DNTT	Bottom-contact	NA	PFBT treatment	10	14
C_{10}-DNTT	Top-contact	Monolayer	vdW contact	59.4	This work

Supplementary Table 2| Performance comparison of low-voltage OTFT technologies.

Dielectric	Material	Thickness (nm)	L/W (μm/μm)	V_{dd} (V)	SS (mV/ dec)	I_{on}/I_{off}	g_m (μS μm^{-1})	Ref.
TiO_2+SAM	DNTT	30	25/300	-1	77	$2.1×10^7$	$2.4×10^{-2}$	15
AlO_X+SAM	DPA	27	NA/6.8	-3	66	$1.1×10^4$	$1.2×10^{-2}$	16
AlO_X+SAM	DPh-DNTT	20	8/200	-3	62	$3.2×10^7$	$1.7×10^{-1}$	13
AlO_X+SAM	DPh-DNTT	20	8/200	-3	68	$6.7×10^6$	$1.8×10^{-1}$	13
AlO_X+SAM	DPh-DNTT	NA	0.6/100	-3	66	$2×10^7$	$1.1×10^0$	14
AlO_X+SAM	C_8-DNBDT-NW	Bilayer	3/750	-10	NA	$4.9×10^4$	$2.5×10^{-4}$	12
AlO_X+SAM	DNTT	20	50/500	-2	NA	$4.1×10^4$	$6.0×10^{-3}$	17
HfO_2+SAM	Pentacene	50	20/1000	-3	75	$1.5×10^5$	$5.4×10^{-3}$	18
AlO_X+PVP	TES-ADT	50	100/1500	-1	85	$5.2×10^4$	$2.0×10^{-3}$	19
PVC	C_8-BTBT	20	40/1400	-3	60.2	$3.5×10^5$	$1.3×10^{-4}$	20
Copolymer	Pentacene	50	30/240	-3	220	$3.7×10^4$	$1.8×10^{-3}$	21
PVDF	PTDPPTFT4	NA	50/1000	-5	120	$1.0×10^3$	$2.0×10^{-3}$	22
PVP	PTDPPTFT4	NA	50/1000	-5	110	$5.4×10^4$	$6.7×10^{-5}$	23
Polymer	C60	50	200/1000	2.5	173	$7.9×10^2$	$1.5×10^{-4}$	24
$Ba_{0.7}Sr_{0.3}TiO_3$	Pentacene	40	100/2000	-2.5	140	$2.6×10^3$	$3.1×10^{-3}$	25
HaLaO	Pentacene	70	80/2000	-2	78	$1.2×10^5$	$5.0×10^{-3}$	26
EDL	C_8-BTBT	NA	100/21000	-5	NA	$1.6×10^1$	$5.3×10^{-6}$	27
EDL	$P(T_0T_0TT_{16})$	NA	2.5/1000	-1	70	$5.8×10^3$	$1.5×10^{-2}$	28
HZO	C_{10}-DNTT	Monolayer	5/180	-1	58.6	$2.0×10^8$	$4.1×10^{-1}$	This work

Supplementary Table 3| Voltage gain comparison of inverters based on different semiconductors.

Materials	V_{dd} (V)	Gain (V/V)	Ref.
IGZO	2	220	29
ITO	0.5/2.5	178/476	30
MoS_2	10	155	31
MoS_2	5	60	32
WSe_2	5.5	340	33
WSe_2	3	12	34
$MoTe_2$	1/2	18/29	35
BP	2	13	36
$MoS_2/MoTe_2$	0.5/1	7.7/33.3	37
C_8-BTBT	2	260	20
DNTT	2	496	17
Pentacene	1.5/2.5	574/478	38
DNTT	3	11	39
DNTT	2	1950	40
Pentacene	4/7	500/1600	41
DPh-BTBT	1	180	42
organic	20	6400	43
CNT	3	290	44
CNT	6	25	45
CNT	5	16	46
CNT	2	30	47
C_{10}-DNTT	-1/-2/-3	4069/6621/11220	This work
C_{10}-DNTT	-1/-2/-3	4825/7690/9762	This work

Supplementary Table 4| Performance comparison of amplifiers for detecting biosignals.

TFT Type	TFT Scale	Circuit topology	Biosignal	V_{dd} (V)	Battery Power	Gain (V/V)	SNR (dB)	Ref.
OFET	4	2-stage inverter	Rat's ECG	2	-	~100	~36	17
OFET	4	2-stage inverter	Pulse	30	-	~10	-	48
OFET	7	1-stage differential amplifiers	ECG	4	-	~16	34	49
IGZO TFT	4	1-stage differential amplifier	HR	10	-	~13	-	50
Dual Gate IGZO TFT	5	1-stage differential amplifier	ECG	10	-	~60	-	51
IGZO TFT	10	Differential Amplifier	ECG	10	-	~32	-	52
CNT-FET	4	1-stage inverter	ECG	10	-	>60	-	53
OFET	2	1-stage inverter	Pulse	1	Yes	~900	40.4	This work
OFET	2	1-stage inverter	ECG	1	Yes	~324	42	This work

Supplementary Movie 1| ECG monitoring by sub-thermionic amplifier and commercial equipment (Prince 180B by Heal Force).

Supplementary References:

1. Cao W, Banerjee K. Is negative capacitance FET a steep-slope logic switch? *Nature Communications* 2020, **11**(1)**:** 196.

2. Zhao Y, Li L, Peng Y, Li Q, Yang G, Chuai X, *et al.* Surface potential-Based Compact Model for Negative Capacitance FETs Compatible for Logic Circuit: with Time Dependence and Multidomain Interaction. 2019 IEEE International Electron Devices Meeting (IEDM); 2019 7-11 Dec. 2019; 2019. p. 7.5.1-7.5.4.

3. Hayashi T, Take N, Tamura H, Sekitani T, Someya T. Alternating current admittance of DNTT-based metal-insulator-semiconductor capacitors. *J Appl Phys* 2014, **115**(9)**:** 093702.

4. Yang SJ, Park K-T, Im J, Hong S, Lee Y, Min B-W, *et al.* Ultrafast 27 GHz cutoff frequency in vertical WSe2 Schottky diodes with extremely low contact resistance. *Nature Communications* 2020, **11**(1)**:** 1574.

5. Zubko P, Wojdeł JC, Hadjimichael M, Fernandez-Pena S, Sené A, Luk'yanchuk I, *et al.* Negative capacitance in multidomain ferroelectric superlattices. *Nature* 2016, **534**(7608)**:** 524-528.

6. Saha AK, Sharma P, Dabo I, Datta S, Gupta SK. Ferroelectric transistor model based on self-consistent solution of 2D Poisson's, non-equilibrium Green's function and multi-domain Landau Khalatnikov equations. 2017 IEEE International Electron Devices Meeting (IEDM); 2017 2-6 Dec. 2017; 2017. p. 13.5.1-13.5.4.

7. Rabe KM, Dawber M, Lichtensteiger C, Ahn CH, Triscone J-M. Modern Physics of Ferroelectrics:Essential Background. *Physics of Ferroelectrics: A Modern Perspective*. Springer Berlin Heidelberg: Berlin, Heidelberg, 2007, pp 1-30.

8. Hulea IN, Russo S, Molinari A, Morpurgo AF. Reproducible low contact resistance in rubrene single-crystal field-effect transistors with nickel electrodes. *Appl Phys Lett* 2006, **88**(11)**:** 113512.

9. Stadlober B, Haas U, Gold H, Haase A, Jakopic G, Leising G, *et al.* Orders-of-Magnitude Reduction of the Contact Resistance in Short-Channel Hot Embossed Organic Thin Film Transistors by Oxidative Treatment of Au-Electrodes. *Advanced Functional Materials* 2007, **17**(15)**:** 2687-2692.

10. Darmawan P, Minari T, Xu Y, Li S-L, Song H, Chan M, *et al.* Optimal Structure for High-Performance and Low-Contact-Resistance Organic Field-Effect Transistors Using Contact-Doped Coplanar and Pseudo-Staggered Device Architectures. *Advanced Functional Materials* 2012, **22**(21)**:** 4577-4583.

11. He D, Qiao J, Zhang L, Wang J, Lan T, Qian J, *et al.* Ultrahigh mobility and efficient charge injection in monolayer organic thin-film transistors on boron nitride. *Science Advances* 2017, **3**(9)**:** e1701186.

12. Yamamura A, Watanabe S, Uno M, Mitani M, Mitsui C, Tsurumi J, *et al.* Wafer-scale, layer-controlled organic single crystals for high-speed circuit operation. *Science Advances* 2018, **4**(2)**:** eaao5758.

13. Borchert JW, Peng B, Letzkus F, Burghartz JN, Chan PKL, Zojer K, *et al.* Small contact resistance and high-frequency operation of flexible low-voltage inverted coplanar organic transistors. *Nature Communications* 2019, **10**(1)**:** 1119.

14. Borchert JW, Zschieschang U, Letzkus F, Giorgio M, Weitz RT, Caironi M, *et al.* Flexible low-voltage high-frequency organic thin-film transistors. *Science Advances* 2020, **6**(21)**:** eaaz5156.

15. Jinno H, Yokota T, Matsuhisa N, Kaltenbrunner M, Tachibana Y, Someya T. Low operating voltage organic transistors and circuits with anodic titanium oxide and phosphonic acid self-assembled monolayer dielectrics. *Organic Electronics* 2017, **40:** 58-64.

16. Yang F, Sun L, Han J, Li B, Yu X, Zhang X, *et al.* Low-Voltage Organic Single-Crystal Field-Effect Transistor with Steep Subthreshold Slope. *ACS Applied Materials & Interfaces* 2018, **10**(31)**:** 25871-25877.

17. Sekitani T, Yokota T, Kuribara K, Kaltenbrunner M, Fukushima T, Inoue Y, *et al.* Ultraflexible organic amplifier with biocompatible gel electrodes. *Nature Communications* 2016, **7:** 11425.

18. Acton O, Dubey M, Weidner T, O'Malley KM, Kim T-W, Ting GG, *et al.* Simultaneous Modification of Bottom-Contact Electrode and Dielectric Surfaces for Organic Thin-Film Transistors Through Single-Component Spin-Cast Monolayers. *Advanced Functional Materials* 2011, **21**(8)**:** 1476-1488.

19. Kim SH, Jang M, Yang H, Anthony JE, Park CE. Physicochemically Stable Polymer-Coupled Oxide Dielectrics for Multipurpose Organic Electronic Applications. *Advanced Functional Materials* 2011, **21**(12)**:** 2198-2207.

20. Jiang C, Choi HW, Cheng X, Ma H, Hasko D, Nathan A. Printed subthreshold organic transistors operating at high gain and ultralow power. *Science* 2019, **363**(6428)**:** 719.

21. Ji D, Li T, Zou Y, Chu M, Zhou K, Liu J, *et al.* Copolymer dielectrics with balanced chain-packing density and surface polarity for high-performance flexible organic electronics. *Nature Communications* 2018, **9**(1)**:** 2339.

22. Wang C, Lee W-Y, Kong D, Pfattner R, Schweicher G, Nakajima R, *et al.* Significance of the double-layer capacitor effect in polar rubbery dielectrics and exceptionally stable low-voltage high transconductance organic transistors. *Scientific Reports* 2015, **5**(1)**:** 17849.

23. Wang C, Lee W-Y, Nakajima R, Mei J, Kim DH, Bao Z. Thiol–ene Cross-Linked Polymer Gate Dielectrics for Low-Voltage Organic Thin-Film Transistors. *Chemistry of Materials* 2013, **25**(23)**:** 4806-4812.

24. Moon H, Seong H, Shin WC, Park W-T, Kim M, Lee S, *et al.* Synthesis of ultrathin polymer insulating layers by initiated chemical vapour deposition for low-power soft electronics. *Nature Materials* 2015, **14**(6)**:** 628-635.

25. Wang Z, Ren X, Leung CW, Shi S, Chan PKL. A UV-ozone treated amorphous barium–strontium titanate dielectric thin film for low driving voltage flexible organic transistors. *Journal of Materials Chemistry C* 2013, **1**(24)**:** 3825-3832.

26. Chang MF, Lee PT, McAlister SP, Chin A. Low Subthreshold Swing HfLaO/Pentacene Organic Thin-Film Transistors. *IEEE Electron Device Letters* 2008, **29**(3)**:** 215-217.

27. Dai S, Chu Y, Liu D, Cao F, Wu X, Zhou J, *et al.* Intrinsically ionic conductive cellulose nanopapers applied as all solid dielectrics for low voltage organic transistors. *Nature Communications* 2018, **9**(1)**:** 2737.

28. Herlogsson L, Crispin X, Tierney S, Berggren M. Polyelectrolyte-Gated Organic Complementary Circuits Operating at Low Power and Voltage. *Advanced Materials* 2011, **23**(40)**:** 4684-4689.

29. Lee S, Nathan A. Subthreshold Schottky-barrier thin-film transistors with ultralow power and high intrinsic gain. *Science* 2016, **354**(6310)**:** 302.

30. Li S, Tian M, Gao Q, Wang M, Li T, Hu Q, *et al.* Nanometre-thin indium tin oxide for advanced high-performance electronics. *Nature Materials* 2019, **18**(10)**:** 1091-1097.

31. Dai Z, Wang Z, He X, Zhang X-X, Alshareef HN. Large-Area Chemical Vapor Deposited MoS2 with Transparent Conducting Oxide Contacts toward Fully Transparent 2D Electronics. *Advanced Functional Materials* 2017, **27**(41)**:** 1703119.

32. Wachter S, Polyushkin DK, Bethge O, Mueller T. A microprocessor based on a two-dimensional semiconductor. *Nature Communications* 2017, **8:** 14948.

33. Kong L, Zhang X, Tao Q, Zhang M, Dang W, Li Z, *et al.* Doping-free complementary WSe2 circuit via van der Waals metal integration. *Nature Communications* 2020, **11**(1)**:** 1866.

34. Tosun M, Chuang S, Fang H, Sachid AB, Hettick M, Lin Y, *et al.* High-Gain Inverters Based on WSe2 Complementary Field-Effect Transistors. *ACS Nano* 2014, **8**(5)**:** 4948-4953.

35. Lim JY, Pezeshki A, Oh S, Kim JS, Lee YT, Yu S, *et al.* Homogeneous 2D MoTe2 p–n Junctions and CMOS Inverters formed by Atomic-Layer-Deposition-Induced Doping. *Advanced Materials* 2017, **29**(30)**:** 1701798.

36. Chen L, Li S, Feng X, Wang L, Huang X, Tee BCK, *et al.* Gigahertz Integrated Circuits Based on Complementary Black Phosphorus Transistors. *Advanced Electronic Materials* 2018, **4**(9)**:** 1800274.

37. Pezeshki A, Hosseini Shokouh SH, Jeon PJ, Shackery I, Kim JS, Oh I-K, *et al.* Static and Dynamic Performance of Complementary Inverters Based on Nanosheet α-MoTe2 p-Channel and MoS2 n-Channel Transistors. *ACS Nano* 2016, **10**(1)**:** 1118-1125.

38. Liu J, Gao X, Xu J, Ruotolo A, Wang S. Flexible Low-Power Organic Complementary Inverter Based on Low- ${k}$ Polymer Dielectric. *IEEE Electron Device Letters* 2017, **38**(10)**:** 1461-1464.

39. Yokota T, Kajitani T, Shidachi R, Tokuhara T, Kaltenbrunner M, Shoji Y, *et al.* A few-layer molecular film on polymer substrates to enhance the performance of organic devices. *Nature Nanotechnology* 2018, **13**(2)**:** 139-144.

40. Kondo M, Uemura T, Matsumoto T, Araki T, Yoshimoto S, Sekitani T. Ultraflexible and ultrathin polymeric gate insulator for 2 V organic transistor circuits. *Applied Physics Express* 2016, **9**(6)**:** 061602.

41. Petritz A, Wolfberger A, Fian A, Griesser T, Irimia-Vladu M, Stadlober B. Cellulose-Derivative-Based Gate Dielectric for High-Performance Organic Complementary Inverters. *Advanced Materials* 2015, **27**(46)**:** 7645-7656.

42. Zschieschang U, Bader VP, Klauk H. Below-one-volt organic thin-film transistors with large on/off current ratios. *Organic Electronics* 2017, **49:** 179-186.

43. Raiteri D, Lieshout Pv, Roermund Av, Cantatore E. Positive-Feedback Level Shifter Logic for Large-Area Electronics. *IEEE Journal of Solid-State Circuits* 2014, **49**(2)**:** 524-535.

44. Lei T, Shao L-L, Zheng Y-Q, Pitner G, Fang G, Zhu C, *et al.* Low-voltage high-performance flexible digital and analog circuits based on ultrahigh-purity semiconducting carbon nanotubes. *Nature Communications* 2019, **10**(1)**:** 2161.

45. Tang J, Cao Q, Tulevski G, Jenkins KA, Nela L, Farmer DB, *et al.* Flexible CMOS integrated circuits based on carbon nanotubes with sub-10 ns stage delays. *Nature Electronics* 2018, **1**(3)**:** 191-196.

46. Sun D-m, Timmermans MY, Tian Y, Nasibulin AG, Kauppinen EI, Kishimoto S, *et al.* Flexible high-performance carbon nanotube integrated circuits. *Nature Nanotechnology* 2011, **6**(3)**:** 156-161.

47. Zhang H, Xiang L, Yang Y, Xiao M, Han J, Ding L, *et al.* High-Performance Carbon Nanotube Complementary Electronics and Integrated Sensor Systems on Ultrathin Plastic Foil. *ACS Nano* 2018, **12**(3)**:** 2773-2779.

48. Wang S, Xu J, Wang W, Wang G-JN, Rastak R, Molina-Lopez F, *et al.* Skin electronics from scalable fabrication of an intrinsically stretchable transistor array. *Nature* 2018, **555:** 83.

49. Sugiyama M, Uemura T, Kondo M, Akiyama M, Namba N, Yoshimoto S, *et al.* An ultraflexible organic differential amplifier for recording electrocardiograms. *Nature Electronics* 2019, **2**(8)**:** 351-360.

50. Zulqarnain M, Stanzione S, Steen JPJVD, Gelinck GH, Myny K, Abdinia S, *et al.* A 52 μW Heart-Rate Measurement Interface Fabricated on a Flexible Foil with A-IGZO TFTs. ESSCIRC 2018 - IEEE 44th European Solid State Circuits Conference (ESSCIRC); 2018 3-6 Sept. 2018; 2018. p. 222-225.

51. Zulqarnain M, Stanzione S, Steen JPJvd, Gelinck GH, Myny K, Cantatore E. A Low Power Time Domain ECG Interface Based on Flexible a-IGZO TFTs. 2019 IEEE 8th International Workshop on Advances in Sensors and Interfaces (IWASI); 2019 13-14 June 2019; 2019. p. 205-209.

52. Garripoli C, Steen JPJvd, Torricelli F, Ghittorelli M, Gelinck GH, Roermund AHMV, *et al.* Analogue Frontend Amplifiers for Bio-Potential Measurements Manufactured With a-IGZO TFTs on Flexible Substrate. *IEEE Journal on Emerging and Selected Topics in Circuits and Systems* 2017, **7**(1)**:** 60-70.

53. Koo JH, Jeong S, Shim HJ, Son D, Kim J, Kim DC, *et al.* Wearable Electrocardiogram Monitor Using Carbon Nanotube Electronics and Color-Tunable Organic Light-Emitting Diodes. *ACS Nano* 2017, **11**(10)**:** 10032-10041.
