# Supplementary material for: Sub-thermionic, ultra-high-gain organic transistors and circuits
Source: Nat Commun. 2021 Mar 26;12:1928. doi: 10.1038/s41467-021-22192-2 (PMC7997979; doi:10.1038/s41467-021-22192-2)
Supplement: Supplementary file 3 — Description of Additional Supplementary Files [file 41467_2021_22192_MOESM3_ESM.pdf]

Description of Additional Supplementary Files

File Name: Supplementary Movie 1
Description: ECG monitoring by sub-thermionic amplifier and commercial equipment (Prince 180B by Heal Force).
